# Long Intergenic Non-Coding RNA 00511 (LINC00511) Genetic Variations and Haplotypes in Breast Cancer: A Case-Controlled Study and Bioinformatics Analysis

**DOI:** 10.3390/ijms26199328

**Published:** 2025-09-24

**Authors:** Shorouk Eldash, Eman F. Sanad, Reham A. A. Elshimy, Ahmad A. Hady, Dina Nada, Nadia M. Hamdy

**Affiliations:** 1 Pharmacology and Biochemistry Department, Faculty of Pharmacy, The British University in Egypt (BUE), El-Sherouk, Cairo 11837, Egypt; 2 Health Research Center of Excellence, Drug Research and Development Group, The British University in Egypt (BUE), El-Sherouk, Cairo 11837, Egypt; 3 Biochemistry Department, Faculty of Pharmacy, Ain Shams University, Abassia, Cairo 11566, Egypt; 4 Clinical and Chemical Pathology Department, National Cancer Institute, Cairo University, Cairo 11796, Egypt; 5 Department of Clinical Oncology and Nuclear Medicine, Faculty of Medicine, Mansoura University, Mansoura 35516, Dakahlia, Egypt

**Keywords:** bioinformatics, breast cancer (BC), ceRNA, genotyping, LINC00511, lncRNA, SNPs, haplotype, prognosis

## Abstract

Long intergenic non-coding RNA 00511 (LINC00511) has been involved in the development of several types of cancer including breast cancer (BC). Several single nucleotide polymorphisms (SNPs) can be found in the genomic regions of long non-coding RNAs (lncRNAs) and are associated with the tumorigenesis of many cancers. The objective of the current study is to assess whether LINC00511 SNPs (rs11657109, rs9906859, rs17780195, rs1558535, and rs4432291) could be related with BC incidence in the Egyptian population. Five SNPs of LINC00511 were analyzed in a case–control study of 267 BC cases and 150 controls. Logistic regression analysis was used to test the association between LINC00511 SNPs and BC incidence. We found that the TT genotype of rs11657109 significantly increased BC incidence (OR: 2.177, 95%CI: 1.260–3.763) and this SNP was associated with high incidence of luminal A BC specifically using different genetic models. Haplotype (A_09_ A_91_ A_35_ G_95_ T_59_) was strongly associated with an increased BC incidence as it was totally absent in controls. These findings suggest that LINC00511 SNP rs11657109 is associated with BC susceptibility in the Egyptian population.

## 1. Introduction

Breast Cancer (BC) is the most frequently diagnosed cancer worldwide. In 2022, there were approximately 2.3 million newly diagnosed cases and 666,000 deaths attributed to BC [1]. According to the Global Cancer Observatory (GLOBOCAN); Cancer Tomorrow prediction tool, BC cases are anticipated to increase by more than 55% by 2050 [2]. A variety of factors have been determined to affect the risk of BC such as age, family history, pregnancy, breast feeding, menopausal status and gene mutations [3]. In case of BC, mammography can be used as a screening method in addition to ultrasound examination and needle biopsy which can aid in the diagnosis of BC [4]. It has been reported that mastectomy and chemotherapy have significantly increased the survival of BC patients [5]. The five-year survival rate for BC is 99% or greater when diagnosed at early localized stage while it drops to 32% when BC is diagnosed at late stages [6].

Non-coding (nc) RNAs have a variety of ways to control how genes are expressed [7,8,9]. Long non coding RNAs (lncRNAs) are transcripts of more than 200 nucleotides that are not translated into proteins [10]. They can modulate gene expression by sponging microRNAs or by interacting with DNA and proteins to influence chromatin structure [11,12]. LncRNAs have been implicated in developmental processes and numerous diseases, particularly cancer [13,14]. Epigenetic regulation, recognized as a hallmark of cancer, affects lncRNA expression. The majority of abnormally expressed lncRNAs or their related downstream genes have an effect on cellular proliferation and apoptotic pathways which are specific to cancer [15,16,17,18,19,20,21,22,23]. Epigenetic mechanisms such as DNA methylation, histone modifications, and chromatin remodeling also interact with environmental factors to shape gene expression and phenotype [24,25,26,27]. Importantly, gene expression in eukaryotes is tightly regulated in a tissue- and stage-specific manner [28,29,30,31,32].

Long intergenic non-coding RNAs (lincRNAs) are lncRNAs that are transcribed from the intergenic regions [33]. It has been determined that approximately 50% of lncRNAs are lincRNAs. They are transcribed by RNA polymerase II and have the same splicing, capping, and polyadenylation possibilities as mRNAs [34]. Long intergenic non-coding RNA 00511 (LINC00511) is a 2265 bp oncogene that is located on chromosome 17q24.3 [35]. Dysregulation of LINC00511 has been proved in a number of cancers such as hepatocellular carcinoma, BC, lung cancer, tongue squamous cell carcinoma, renal cell cancer, papillary thyroid carcinoma, osteosarcoma, pancreatic cancer, gastric cancer, glioma, ovarian cancer, colorectal cancer, and cervical cancer [36]. It has been reported that LINC00511 induces the growth of BC cells via acting as competing endogenous RNA (ceRNA) for miR-185-3p and targeting the E2F1 protein, which binds with the Nanog promoter region to activate its transcription, in addition to transcriptional control of downstream genes [35,37]. In addition, Shi et al. have found that LINC00511 can sponge miR-150, resulting in regulation of the expression of Matrix Metallopeptidase 13 (MMP13) and inducing BC cell migration [38].

In the human genome, one of the most prevalent types of genetic variations are single nucleotide polymorphisms (SNPs) [39]. SNPs are single base substitutions at particular genomic locations that may occur in every 100–300 bases [40,41]. Single nucleotide polymorphisms (SNPs) are responsible for the occurrence of numerous malignancies, making them markers for cancer prognosis or drug resistance [42,43,44,45,46]. Finding an association between LINC RNA SNPs and disease mechanisms is a crucial research gap that has to be filled in order to link particular variants to changes in cancer prevalence, progression, or remission [47]. It has been reported that LINC00511 SNPs rs11657109, rs17780195, and rs9906859 may reduce the risk of BC in the Han Chinese population [48].

The current study aims to investigate the association between LINC00511 SNPs rs11657109, rs9906859, rs17780195, rs1558535, and rs4432291 and BC risk in the Egyptian population after they have been studied in the Han Chinese population [48]. Second, it aims to explore the associations between LINC00511 SNPs and the ER, PR, and HER-2 status of BC patients. Third, it aims to study the associations between LINC00511 SNPs, tumor grade, tumor stage, lymph node metastasis (LNM), and BC molecular subtypes.

## 2. Results

### 2.1. In Silico Search and Bioinformatics Analysis Results (Accessed on 21 April 2025)

#### 2.1.1. Differential Gene Expression (DGE) Analysis Results in BRCA (Figure 1a)

**Figure 1:**
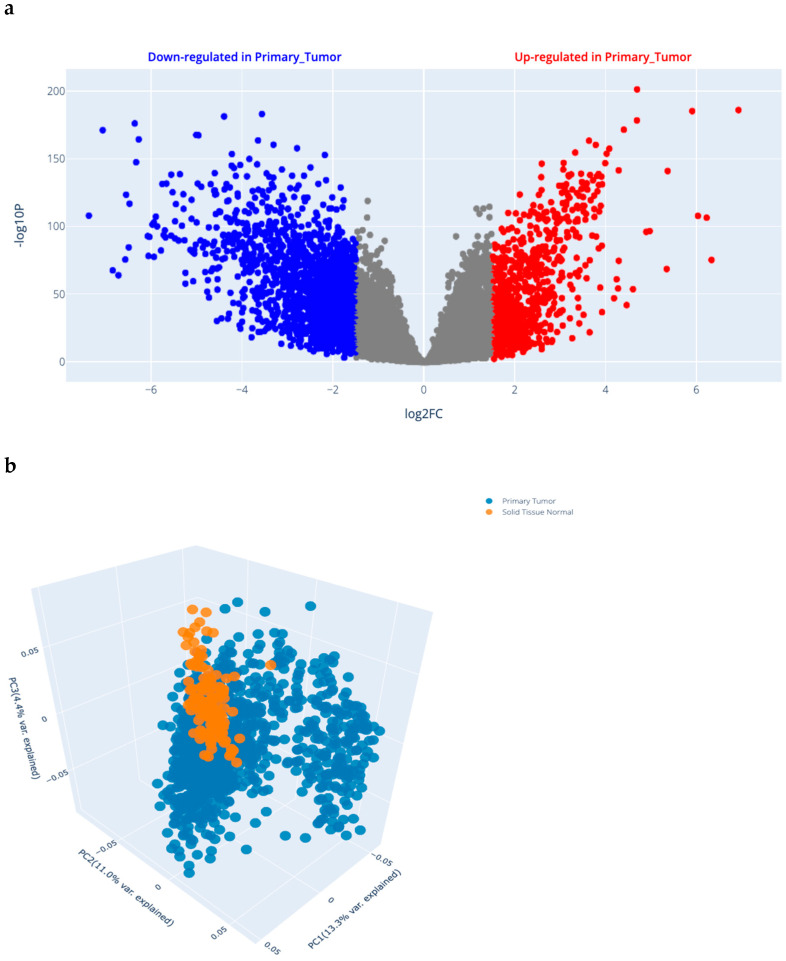
(**a**) Volcano scatter plot for Primary_Tumor vs. Solid_Tissue_Normal signature; log2-fold changes and statistical significance of each gene calculated by performing differential gene expression analysis (info: log-transformed data, base 2 exponentiation is applied); genes with logFC > 1.5 and *p*-value < 0.05 in red, and genes with logFC < −1.5 and *p*-value < 0.05 in blue. (**b**) Three-dimensional QC scatter plot for samples using 2500 genes having largest variance; each point represents gene expression sample; samples with similar gene expression profiles are closer in three-dimensional space.

Xena DGE analysis was run using gene expression data for primary tissue vs. solid normal tissue, and the gene expression dataset was IlluminaHiSeq. Gene expression data was normalized (Z normalization), applied to convert raw read counts into informative measures of gene expression and to remove factors that affect the analysis. The PCA 3D QC scatter plot for samples is presented in Figure 1b. DEGs, (16,701) using limma_voom with their Log2FC, average expression, and *p*-value, are attached as a Supplementary Excel File S1. Supplementary Table S1 addresses the top 10 upregulated genes from DGE analysis.

#### 2.1.2. Selected SNP Criteria

The SNP information report is shown in Table S2 [49,50] https://www.ncbi.nlm.nih.gov/snp/, https://www.ensembl.org/ (accessed on 21 April 2022).

### 2.2. Participants Demographic and Clinical Data

This study included 417 participants (267 BC patients and 150 healthy controls). As shown in Table S3, there was a high risk of BC in post-menopausal women (*p* < 0.001, OR: 2.785, 95%CI: 1.815–4.271). The likelihood of BC increased with the increase in the number of pregnancies (3 pregnancies, *p* = 0.001, OR: 3.518, 95%CI: 1.653–7.485; ≥4 pregnancies, *p* < 0.001, OR: 18.941, 95%CI: 7.035–50.997).

### 2.3. The Association Between LINC00511 SNPs and BC Susceptibility Using Different Genetic Models

The association between LINC00511 SNPs and BC susceptibility was analyzed using different genetic models, and the results are shown in Table 1. SNP rs11657109 exhibited significant association with BC risk. In the codominant model, the TT genotype of SNP rs11657109 was significantly associated with increased BC risk (*p* = 0.005, OR: 2.177, 95%CI: 1.260–3.763). Therefore, LINC00511 SNP rs11657109 could be considered as an important marker for the occurrence of BC. No significant associations with BC susceptibility were found in the remaining SNPs. Although LINC00511 SNP rs9906859 reached nominal significance (*p* < 0.05), it did not meet the stringent pre-specified threshold after correcting for multiple comparisons and was therefore not considered statistically significant.

### 2.4. Alleles Frequencies of the Five SNPs in All the Study Subjects and Their Association with BC

As shown in Table 2, the T allele of SNP rs11657109 was found to be associated with increased risk of BC (*p* = 0.003, OR: 1.454, 95%CI: 1.162–2.056).

### 2.5. Stratification Analysis of the Relationship Between LINC00511 SNPs and BC Susceptibility Using Different Genetic Models

Study subjects were stratified by reproductive factors: age, menopausal status, and number of pregnancies. The relationship between LINC00511 SNPs and BC risk was tested using different genetic models.

#### 2.5.1. Stratification Analysis of the Relationship Between LINC00511 SNPs and BC Susceptibility Using the Codominant Model

As demonstrated in Table S4, the TT genotype of rs11657109 significantly increased the risk of BC in the following subgroups; pre-menopausal (*p* = 0.003, OR: 3.004, 95%CI: 1.444–6.248) and number of pregnancies <3 (*p* = 0.009, OR: 2.827, 95%CI: 1.289–6.199). In addition, the AT genotype significantly increased the susceptibility of BC in women having less than three pregnancies (*p* = 0.005, OR: 2.742, 95%CI: 1.353–5.555). Therefore, LINC00511 SNP rs11657109 could be considered as a prognostic marker for the occurrence of BC, specifically in premenopausal women who have had less than three pregnancies.

#### 2.5.2. Stratification Analysis of the Relationship Between LINC00511 SNPs and BC Susceptibility Using the Dominant Model

Table S5 shows that the AT+TT genotypes of the SNP rs11657109 were associated with increased BC risk in women having a number of pregnancies < 3 (*p* = 0.002, OR: 2.773, 95%CI: 1.432–5.370).

#### 2.5.3. Stratification Analysis of the Relationship Between LINC00511 SNPs and BC Susceptibility Using the Recessive Model

As shown in Table S6, no significant associations with BC susceptibility were found in the remaining SNPs.

#### 2.5.4. Stratification Analysis of the Relationship Between LINC00511 SNPs and BC Susceptibility Using the Over-Dominant Model

As shown in Table S7, the CT genotype in the over-dominant model of the SNP rs9906859 was associated with increased BC risk in post-menopausal women (*p* = 0.009, OR: 3.057, 95%CI: 1.322–7.068).

### 2.6. The Associations of LINC00511 SNPs with ER, PR, and HER-2 Status of BC Patients

We also tested the associations of LINC00511 SNPs and the ER, PR, and HER-2 status of BC patients. The results are shown in Table S8. No significant associations were found between LINC00511 SNPs and the ER, PR, and HER-2 status of BC patients.

### 2.7. The Association Between LINC00511 SNPs and Tumor Stage

We analyzed the association between LINC00511 SNPs and tumor stage, as demonstrated in Table S9. We divided the patients into two groups; the first group is the low tumor stages, which included patients with tumor stage I and II, and the second group is the high tumor stages, which included patients with tumor stages III and IV. A comparison was carried out between the two groups. No significant associations were found between LINC00511 SNPs and the tumor stage of BC patients.

### 2.8. The Association Between LINC00511 SNPs and Lymph Node Metastasis

We also divided the patients into two groups—the first group without lymph node metastasis and the second group with lymph node metastasis—and then we investigated the association between LINC00511 SNPs and lymph node metastasis. As demonstrated in Table S10, there were no significant associations between LINC00511 SNPs and the lymph node metastasis of BC patients.

### 2.9. The Association Between LINC00511 SNPs and Tumor Grade

We investigated the association between LINC00511 SNPs and tumor grade by comparing BC patients with a low tumor grade and those with a high tumor grade. We did not find any significant association between LINC00511 SNPs and tumor grade, as shown in Table S11.

### 2.10. The Association Between BC Molecular Subtypes and LINC00511 SNPs, Relative to Controls

In the current study, the most common subtype of BC was luminal A (*n* = 175), followed by luminal B (*n* = 64) and HER-2 positive (*n* = 16) and the least common subtype was triple-negative BC (TNBC) (*n* = 12). As shown in Table 3, the AT+TT genotypes in the dominant model of the SNP rs11657109 were associated with high risk for the luminal A BC subtype (*p* = 0.003, OR = 2.087, 95%CI = 1.280–3.405). Therefore, LINC00511 SNP rs11657109 could be considered as a prognostic marker for the luminal A BC subtype.

### 2.11. The Association Between LINC00511 SNPs and BC Molecular Subtypes “TNBC and Triple Positive BC”

We analyzed the association between LINC00511 SNPs and the BC molecular subtypes “TNBC and triple positive BC”. As shown Table S12, we did not find any significant association between LINC00511 SNPs and TNBC or triple-positive BC.

### 2.12. The Association Between LINC00511 SNPs and BC Molecular Subtypes “Luminal B and Non-Luminal B BC”

We also investigated the association between LINC00511 SNPs and the BC molecular subtypes “luminal B and non-luminal B BC”, but we did not find any significant association between LINC00511 SNPs and luminal B and non-luminal B BC, as shown in Table S13.

### 2.13. Haplotype Analysis of the Five SNPs in LINC00511

Haplotype analysis was performed for LINC00511 SNPs (rs11657109, rs4432291, rs1558535, rs17780195, and rs9906859) to examine their combined effects, and the results are shown in Table 4. Haplotype (A_rs11657109_ A_rs4432291_ A_rs1558535_ A_rs17780195_ T_rs9906859_) had the highest frequency of all haplotypes among both cases and controls (23.76% and 36.67%, respectively). Haplotype (A_rs11657109_ A_rs4432291_ A_rs1558535_ A_rs17780195_ T_rs9906859_) was significantly associated with decreased risk of BC (*p* = 0.003, OR: 0.617, 95%CI: 0.450–0.846). On the other hand, haplotypes (A_rs11657109_ A_rs4432291_ A_rs1558535_ G_rs17780195_ T_rs9906859_) and (T _rs11657109_ G_rs4432291_ T_rs1558535_ A_rs17780195_ C_rs9906859_) were significantly associated with increased BC risk (*p* = <0.001, OR: NA, 95%CI: NA) and (*p* = 0.001, OR: 1.945, 95%CI: 1.298–2.915), respectively. (A _rs11657109_ A_rs4432291_ A_rs1558535_ G_rs17780195_ T_rs9906859_) is of special interest, as it was totally absent in the control group.

### 2.14. Multifactor Dimensionality Reduction (MDR) Using a Three-Way Split Internal Validation Approach

The MDR approach was utilized to reduce the dimensionality of the problem by identifying the most informative combinations of SNPs that contribute to the outcome of interest. Table S14 showed that the best-performing model with three SNPs (SNP2, SNP4, and SNP5) showed the highest accuracy in all sets, especially with a validation accuracy of 72.1%. This indicates that the interactions between these three SNPs contribute more to predicting the outcome than any one or two-SNP models.

### 2.15. Post Hoc Epistasis Analysis After MDR Model Fit with a Three-Way Split

**Figure 2:**
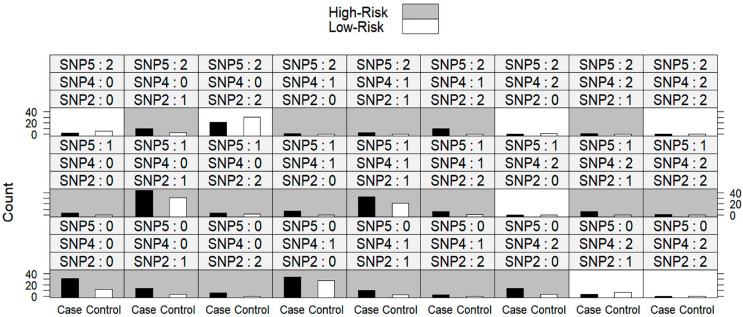
Post hoc epistasis analysis after MDR model fit with three-way split for LINC00511 SNPs for BC cases (*n* = 267) and controls (*n* = 150). SNP2 is rs4432291, SNP4 is rs17780195 and SNP5 is rs9906859. Genotypes are coded ‘0’ for the wild-type, ‘1’ for heterozygous, and ‘2’ for homozygous. Grey bars denote high risk and white bars denote low risk.

Post hoc comparison analysis (Figure 2) showed that individuals who are all-heterozygous or all-homozygous for the wild allele in all studied loci are at high risk of BC. In addition, individuals who are homozygote mutants and heterozygotes or who are heterozygotes and homozygote mutants for rs4432291 and rs17780195, respectively (i.e., [AA/AG/CT or TT] or [AG/GG/CT or TT]), are at high risk of BC as well, suggesting that the interaction between rs443229 and rs17780195 is more predictive of BC risk. Moreover, individuals who are heterozygote and homozygote mutants for rs4432291 and rs9906859, respectively, are at high risk of BC regardless of SNP4’s value.

### 2.16. Linkage Disequilibrium and Pairwise Correlation Coefficient

#### 2.16.1. Population’s Linkage Disequilibrium

The NIH-LD pair tool reported that LINC00511 SNPs in the African population were in strong linkage disequilibrium according to D’ values, as appears in Table S15, except the moderate linkage disequilibrium between rs11657109 and rs4432291. However, correlation coefficient (r^2^) values indicate that they are low-to-moderately correlated, which may be attributed to differences in allele frequencies between SNPs in the population [51] (https://ldlink.nih.gov/?tab=ldpair (accessed on 29 June 2024)).

#### 2.16.2. Testing for Linkage Disequilibrium and Pairwise Correlation Coefficient with Haplotypes

**Figure 3:**
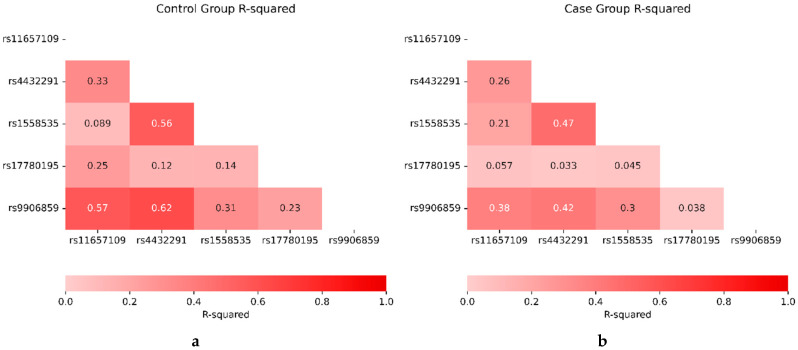
R-squared comparison between control and case groups. Haplotype block analysis between LINC00511 SNPs (rs11657109, rs17780195, rs9906859, rs4432291, and rs1558535) calculated linkage disequilibrium in pairs in controls (*n* = 150) (**a**) and BC patients (*n* = 267) (**b**).

The collected genotype data was used to evaluate linkage disequilibrium. For each pair of LINC00511 polymorphism loci under investigation, pairwise standardized linkage disequilibrium (D’) and haplotype analysis were computed to assess the relationship between LINC00511 SNPs and BC susceptibility. Figure 3 compared the R-squared values for five SNPs between a control group and a case group. In genetic studies, R-squared is used to assess the correlation between two SNPs and measures the degree to which genetic variation at one SNP can predict variation at another. Higher values of R-squared indicate stronger correlations, meaning the SNPs tend to be inherited together, while lower values indicate weaker or no correlation.

In the control group (left panel), several SNP pairs showed moderate to strong correlations. For instance, SNP rs11657109 and rs9906859 exhibited an R-squared value of 0.57, implying a relatively strong relationship between them in the control population. Similarly, rs4432291 and rs9906859 showed an even higher R-squared of 0.62, indicating that these two SNPs are often inherited together. Other SNP pairs, such as rs11657109 and rs1558535, showed lower R-squared values (0.089), indicating weaker linkage between them in the control group.

In contrast, the case group (right panel) presented noticeable differences in linkage patterns. While some SNP pairs, like rs4432291 and rs9906859, still maintained a relatively strong correlation (R^2^ = 0.42), the overall linkage between SNPs was weaker compared to the control group. For example, the correlation between rs11657109 and rs9906859 dropped to 0.38, indicating a reduction in the strength of linkage disequilibrium between these two SNPs in the case group. This weakening of correlations suggests that in individuals with BC, these SNPs are inherited in a more independent manner, possibly due to genetic recombination events or other population-specific factors. The differences in R^2^ between the two groups hint at variations in the underlying genetic architecture, which may be influenced by disease-associated genetic regions or population stratification.

**Figure 4:**
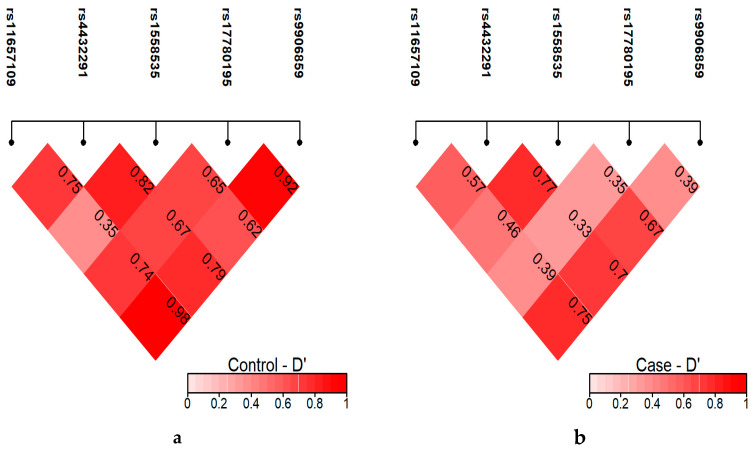
D-prime comparison between control and case groups. Haplotype block analysis between LINC00511 SNPs (rs11657109, rs17780195, rs9906859, rs4432291, and rs1558535) calculated pairwise correlation coefficient in pairs in controls (*n* = 150) (**a**) and BC patients (*n* = 267) (**b**).

Figure 4 focused on D-prime (D’), another statistic used to measure linkage disequilibrium, but with a focus on whether SNPs are co-inherited at the maximum rate possible given their allele frequencies. D-prime values close to 1 indicate that the SNPs are nearly always inherited together, while values closer to 0 suggest weak or no linkage.

In the control group, several SNP pairs demonstrated strong linkage, with some D-prime values approaching 1. For example, rs11657109 and rs9906859 had a D-prime of 0.98, indicating that these two SNPs are almost always inherited together in the control population. Other SNP pairs, such as rs4432291 and rs1558535, also showed strong linkage with a D-prime of 0.82, suggesting that these SNPs are tightly linked in the control group. This high level of linkage across many SNP pairs suggests that recombination events in the control group are relatively rare in these genomic regions, leading to the consistent inheritance of specific SNP combinations.

Pairwise LD analysis showed a strong association in BC group between different pairs, such as rs11657109 and rs9906859 as well as rs4432291 and rs9906859. However, it showed a more fragmented pattern of linkage disequilibrium. For example, the D-prime value between rs11657109 and rs9906859 decreased to 0.75, indicating that while these SNPs were still often inherited together, the strength of their linkage was weaker compared to the control group. Similarly, rs4432291 and rs9906859 exhibited a decrease in D-prime from 0.79 in the control group to 0.70 in the case group. These reductions in D-prime suggest that recombination events or other genetic factors have disrupted the inheritance patterns of these SNPs in the case group.

## 3. Discussion

In the Middle East, Egypt has one of the highest rates of BC with an incidence rate of 48.8/10^5^ [52]. Predicting BC risk is an important topic with a number of factors affecting the precision and applicability of such predictions such as age, pregnancy, menopausal status, and hormonal factors [53]. Proactive monitoring and preventative actions are made possible by the identification of high-risk individuals [54].

The basic characteristics of 267 BC patients and 150 cancer-free controls were analyzed, and we found that there was a high risk of BC in post-menopausal women in the Egyptian population consistent with the findings of Chong et al. in their study on BC in a Han Chinese population [48]. Similarly, Sankar et al. found a strong positive association between post-menopause and increased BC risk where post-menopausal women had a 2.7 times higher risk of BC than pre-menopausal women [55]. The association between post-menopause and specific BC subtypes was analyzed by previous case–case analysis, where it was reported that patients with HER-2 overexpression and TNBC subtypes were more likely to be post-menopausal compared with patients with the luminal A BC subtype [56]. In 2018, Heer et al. documented that there were almost 6.4 million post-menopausal and 645 thousand pre-menopausal BC cases diagnosed globally, with over 130 000 and 490 000 deaths in each group, respectively [57]. On the other hand, a study reported that being post-menopausal was linked to a statistically significant lower risk of BC in general and especially luminal A-like BC [58]. This emphasizes the significance of proactive healthcare in the post-menopausal years [59].

Moreover, we found that the risk of BC increased with the increase in the number of pregnancies (≥3 pregnancies), which is also consistent with the findings of Chong et al. in their study on BC in a Han Chinese population [48]. Similarly, it has been reported that multiple pregnancies increased the risk of BC in Chinese women [60]. In addition, a study found that number of pregnancies showed a positive association with TNBC subtype but a negative association with luminal A BC subtype [61]. However, other studies reported that that an increased number of pregnancies is protective against BC, particularly hormone-positive BC [62,63]. It was also found that having no pregnancies was strongly linked to an increased incidence of the ER+ BC subtype [64]. Similarly, it was also documented that few or no pregnancies at all was linked to a higher risk of the ER+ BC subtype and lower risk of the TNBC subtype [65]. In addition, it was reported that there was an inverse association between number of pregnancies and the luminal A and B BC subtypes [58]. Moreover, few or no pregnancies were linked to increased risk of luminal A, luminal B, and HER-2 subtypes [56]. A study found that the number of pregnancies was inversely correlated with the risk of luminal A, luminal B, and HER-2 BC subtypes but not associated with the risk of the TNBC subtype in African American women [66]. Therefore, from the previous mentioned studies, we suggest that the influence of reproductive factors may differ among different BC subtypes.

Recent researches suggested that ncRNAs are potential players in the development of various cancers [67,68] including BC [5]. LincRNAs offer an exciting new area of genetics that have an important role in cancer [10,69]. LINC00511 has been linked to the development of several cancers including BC [36]. As mentioned before, LINC00511 has been reported to contribute to BC proliferation via different pathways. Additionally, Zhang et al. reported that LINC00511 enhanced the growth of ER-negative BC cells. LINC00511 is a potentially fruitful direction for cancer research [70].

SNPs are a type of genetic variation that may arise anywhere in the genome. Research has demonstrated that SNPs, particularly lncRNAs, can affect the progression of cancer [71]. An SNP may alter the lncRNA’s secondary structure, which may have an impact on the lncRNA’s capacity to bind to proteins, DNA, or other RNAs [72].

The current study is the first study to investigate the association between LINC00511 SNPs and BC risk in the Egyptian population. We tested SNPs rs11657109, rs17780195, rs9906859, rs4432291, and rs1558535. As recorded by NCBI-dbSNP, all of these SNPs are located in the intronic regions [49]. SNPs rs11657109, rs17780195, and rs9906859 were reported to be significantly associated with BC in a Han Chinese population [48].

We explored the alleles’ frequencies of the five SNPs in the study subjects and their association with BC. We found that the distribution of alleles of LINC00511 SNP rs11657109 significantly differed between the control group and BC patients, showing higher incidence of the T allele in BC patients than controls. Thus, the T allele of LINC00511 SNP rs11657109 was associated with increased risk of BC.

In addition, we tested the association between the genotypes of SNPs and BC susceptibility. There was a positive association between SNP rs11657109 and BC risk, as indicated by TT genotype in the codominant model. This result is opposite to what was reported by the research group who studied the same SNPs in the Han Chinese population, which may be attributed to differences in ethnicity between the Egyptian and Chinese populations [48]. In addition, there was no significant effect of SNPs rs9906859 and rs17780195 on BC risk in the Egyptian population, but they were associated with decreased BC risk in the Chinese population, which may also be due to differences in ethnicity between the two populations. The association between the LINC00511 SNP and BC may be attributed to the effect of the SNP on the expression and/or secondary structure of LINC00511 [47]. The association may also be attributed to the fact that this SNP is in high linkage disequilibrium with the truly causative variants [73].

Stratification analysis is important in research as it facilitates the recognition and comprehension of risk factors within a particular population [74]. After subjects’ stratification based on multiple factors, the association of LINC00511 SNP rs11657109 TT genotype persisted in the following subgroups: pre-menopausal women and women with less than three pregnancies. In addition, the AT genotype significantly increased the susceptibility of BC in women having less than three pregnancies. These results suggest that this SNP might be a potential marker for the occurrence of the disease. In addition, LINC00511 SNP rs9906859 CT genotype showed significant association with BC in the subgroup of post-menopausal women, as indicated by the over-dominant model. Interestingly, the association of the rs9906859 CT genotype with BC risk emerged only in the post-menopausal subgroup. This could be a reflection of how post-menopausal hormonal changes affect genetic susceptibility, which could unmask associations that are not visible in the general population. This subgroup-specific effect may potentially be also attributed to post-menopausal women’s gene–environment or epigenetic interactions [75].

Investigating the relationship between SNPs and tumor characteristics such as tumor grade, tumor stage, PR status, and lymph node metastasis yields important information about cancer biology, informs tailored treatment plans, improves risk assessment and screening protocols, helps track the course of the disease, and eventually improves oncology patient outcomes [76,77,78]. Therefore, we performed association analyses among female BC patients, and no significant association was found between SNPs and tumor characteristics. The study in the Han Chinese population found a different result where LINC00511 SNPs (rs4432291, rs1558535, rs17780195, and rs9906859) were associated with the HER-2 status [48].

Molecular subtyping showed that the most common subtypes of BC in the current study in Egypt were luminal A and luminal B. The analysis of the association between LINC00511 SNPs and molecular types of BC showed that the AT+TT genotypes of the LINC00511 SNP rs11657109 were also associated with high risk of luminal A BC, as indicated by the dominant model. Thus, women who have this polymorphism are at high risk for BC development, especially the luminal A subtype, which has the best prognosis due to low expression of cell proliferation marker Ki-67 [79].

Chen et al. reported that analyzing combinations of genetic variants (haplotypes) can provide a more powerful assessment of disease risk compared to studying single SNPs alone [80]. Haplotype analysis was performed for the five LINC00511 SNPs to test their combined effects. It showed that haplotype (A_rs11657109_ A_rs4432291_ A_rs1558535_ A_rs17780195_ T_rs9906859_) was present at the highest frequency in cases and controls and was found to be significantly associated with decreased BC risk in the Egyptian population. This result is opposite to findings of Chong et al., who found that the same haplotype was significantly associated with increased BC risk in Han Chinese population [48]. On the other hand, the risk of BC was increased with haplotypes (A_rs11657109_ A_rs4432291_ A_rs1558535_ G_rs17780195_ T_rs9906859_) and (T_rs11657109_ G_rs4432291_ T_rs1558535_ A_rs17780195_ C_rs9906859_). Interestingly, haplotype (A_rs11657109_ A_rs4432291_ A_rs1558535_ G_rs17780195_ T_rs9906859_) was completely absent from the control group, which means that it may be strongly associated with increased BC susceptibility.

Interaction analysis using MDR was used, and the results indicated that the interaction model becomes more complex, from one SNP to three SNPs. The predictive accuracy improved, particularly in the validation set. This suggests that a multifactor interaction model, especially involving SNP2, SNP4, and SNP5, may be the most reliable for predicting the outcome in this dataset.

Post hoc epistasis analysis was performed to know how specific genetic patterns contribute to risk, paving the way for further exploration of these genetic markers in relation to the outcome of interest. It was found that individuals who are all-heterozygous or all-homozygous for the wild allele in all studied loci are at high risk of BC. Moreover, individuals who are homozygote mutants and heterozygotes or who are heterozygote and homozygote mutants for rs4432291 and rs17780195, respectively, are at high risk of BC as well, suggesting that the interaction between rs443229 and rs17780195 is more predictive of BC risk. Moreover, individuals who are heterozygote and homozygote mutants for rs4432291 and rs9906859, respectively, are at high risk of BC regardless of SNP5’s value, suggesting that the interaction between rs443229 and rs9906859 is also more predictive of BC risk. Hence, by examining combinations, this approach helped to uncover these multi-locus interactions, which might otherwise go undetected in single-SNP analyses.

In this study, pairwise LD analysis was performed. It showed a strong association in the BC group between different pairs, such as rs11657109 and rs9906859 as well as rs4432291 and rs9906859. However, the strength of their linkage was weaker compared to the control group. Overall, the lower D-prime values in the case group indicate that the SNPs are less tightly linked than in the control group, implying that genetic recombination might have occurred more frequently or that specific genetic factors unique to the disease or condition are influencing the inheritance patterns. These results highlight a potential divergence in genetic structure between the two groups, which could be associated with the disease phenotype being studied. Such differences in linkage disequilibrium can provide valuable clues for identifying genetic loci that contribute to disease susceptibility or resistance in the case group.

A key strength of this research is the recruitment of a substantial number of subjects, ensuring the study’s findings can be applied to a more general population. Our study investigated specific LINC00511 SNPs which have been rarely studied, with only one prior publication addressing them in a different population, which highlighted the novelty and relevance of our findings. LINC00511 SNP rs11657109 represents a prospective marker for the prediction of BC risk and may be used as a marker to differentiate between different BC subtypes. Further research and functional studies are currently planned and being carried out by our group to unravel the molecular mechanism behind the association between LINC00511 SNPs and BC. The reported higher BC incidence/risk among women with more than three pregnancies warrants further exploration as well.

Limitations: Even though our findings offer important insights into the association between LINC00511 polymorphisms and BC subtypes, SNPs’ association with therapy (e.g., endocrine therapy, chemotherapy) response data was not available when conducting this study. But, whether these SNPs are predictive of BC treatment outcomes or resistance and whether they are related to specific biological pathways (via Gene Set Enrichment Analysis (GSEA) or DAVID functional annotation) are currently being evaluated with survival outcomes (in progress).

## 4. Materials and Methods

### 4.1. Sample Size and Power of the Study

Sample size calculation was performed using the comparison of prevalence of the GG genotype in rs17780195 SNP between BC patients and normal healthy matched individuals. Calculation was carried out based on the odds ratio (OR) from independent samples in a prospective study using the Fisher exact test, the α-error level was fixed at 0.05, the power was set at 80%, and the case–control ratio was set at 1. As previously published [48], the prevalence of the GG genotype in rs17780195 SNP among normal healthy controls was approximately 64.9%, and the OR between BC cases and healthy controls was approximately 0.398. Accordingly, the minimum optimum sample size should be 85 participants for each SNP group. Sample size calculation was performed using PS: Power and Sample Size Calculations software, version 3.0.11 for MS Windows (William D. Dupont and Walton D., Vanderbilt University, Nashville, TN, USA).

### 4.2. Study Design

The study is a case-controlled retrospective observational study.

### 4.3. Study Participants

#### 4.3.1. Patient Group

Two hundred sixty-seven treatment-naive female Egyptian patients with BC, aged 20–70 years, were recruited from the National Cancer Institute (NCI), New Cairo, Egypt, for this study.

A full history was collected and recorded for the patient group (*n* = 267). The patients were selected according to inclusion and exclusion criteria.

##### Patients’ Inclusion Criteria

This study included female patients aged 20–70 years who visited the NCI for a breast examination and presented with one or more of the following symptoms: a new lump in the breast or underarm; changes in the size, shape, or appearance of a breast; dimpling of breast skin; nipple discharge (from either nipple); and redness or flaky skin in the breast or nipples. BC diagnosis was clinically confirmed by mammogram, breast ultrasound, breast MRI, and a biopsy from the breast.

##### Patients’ Exclusion Criteria

This study excluded males, females aged less than 20 or more than 70, subjects receiving any chemotherapy or radiotherapy or who underwent surgery, patients with blood disorders, any other active cancer diagnosis or blood-borne diseases, and chronic health conditions such as neuronal diseases, respiratory diseases, uterine diseases, kidney diseases, and cirrhosis of the liver. Additionally, patients with prolonged use of corticosteroids or sex hormones and patients with incomplete data or histopathology diagnosis were excluded.

##### Patients Pathological and Clinical Data

The clinical assessment of BC patients’ tumors was carried out at the Pathology Unit, NCI, New Cairo. Patients were clinically confirmed by mammogram, breast ultrasound, breast MRI, a biopsy from the breast, and pathological reports. The BC patients’ hormonal profile, HER-2 status, levels of Ki-67, Carcinoembryonic Antigen (CEA) and Cancer Antigen 15-3 (CA15-3), tumor grade, tumor size, Breast-Imaging Reporting and Data System (BIRADS), tumor-node-metastasis (TNM) staging, American College of Radiology (ACR), LNM, number of pregnancies, height, weight, menopausal status, family history, and non-communicable diseases status were all recorded from patients’ files at the NCI, New Cairo, Egypt. All the included histopathological parameters were derived from the original pathology reports. BIRADS classification is according to the American College of Radiology. Specialized pathologist(s) performed scoring at the NCI Pathology Department. These scorings were made according to standardized protocols, and the pathologist was unaware of the study objectives. According to TNM categorization, patients are categorized into five stages; stage 0 describes non-invasive BC while stages I to IV describe invasive BC. In addition, histopathological grades were determined based on the Nottingham grading system through examining morphologic characteristics such as tubular differentiation, mitotic count, nuclear pleomorphism. Each aspect is given a score ranging from 1 (most favorable) to 3 (least favorable). The combined scores for tumor grades 1, 2, and 3 fall between 3 and 5, 6 and 7, and 8 and 9, respectively.

#### 4.3.2. Control Group

A total of one hundred and fifty healthy female volunteers, not taking any medication or suffering from any disease, with normal kidney functions and liver enzyme levels and absence of any clinical or laboratory evidence of BC, were randomly selected as controls. Control females were recruited during routine checkup examinations for themselves or their relatives.

### 4.4. In Silico Search and Bioinformatics Analysis

#### 4.4.1. Differential Gene Expression of Different Genes from Online Datasets in BC

To retrieve relevant gene expression data, we accessed the UCSC Xena Browser (https://xenabrowser.net) and selected the dataset (TCGA BC (BRCA)). The first variable was phenotypic sample type, as primary tissue vs. solid normal tumor (https://xenabrowser.net/heatmap/), to compare the expression of different genes in available online datasets via the Xena Differential Gene Expression Analysis Pipeline (https://github.com/ucscXena; adapted from the Ma’ayan lab’s Appyter bulk RNA-seq analysis, https://appyters.maayanlab.cloud/#/Bulk_RNA_seq). We also ran a differential gene expression (DGE) analysis and further downstream analyses (http://analysis.xenahubs.net/e08c31d726d85e8e343e534eae6c6232245b1768/). The second variable wasSLC39A11 gene expression. (All websites in Section 4.4.1 and Section 4.12 were accessed on 21 April 2025).

#### 4.4.2. Principal Component Analysis (PCA)

For detecting overarching patterns in high-dimensional data to assess the similarity between biological samples in gene expression studies, we again used the Xena Differential Gene Expression Analysis Pipeline.

### 4.5. LINC00511

The National Center for Biotechnology Information (NCBI) [81] (https://www.ncbi.nlm.nih.gov/nuccore/NR_033876) (accessed on 21 April 2022) defines the gene, and its locus, name. The Human ncRNA Database (GeneCaRNA) [82] (https://www.genecards.org/cgi-bin/carddisp.pl?gene=LINC00511) was accessed to know information about LINC00511 (accessed on 31 March 2024), where aliases for *LINC00511* gene are linc RNA 511 2 3 5; Onco-LncRNA-12 2 3 5; LCA LncRNA 5 3; NONHSAG022655.2 83; Lnc-SLC39A11-1 148; HSALNG0118540 147; and LCAL5.

### 4.6. SNP Selection

The information for chosen SNPs was collected from NCBI-dbSNP [49] at https://www.ncbi.nlm.nih.gov/snp/ and [50] https://www.ensembl.org/ (accessed on 21 April 2022). Minor allele frequency (MAF) for each SNP was obtained from the International Genome Sample Resource (IGSR) in 1000 genomes [50,83] (accessed on 21 April 2022). Based on the prior study by Chong et al. that studied multiple LINC00511 SNPs [48] and following screening for a MAF greater than 0.05, LINC00511 SNPs rs11657109, rs9906859, rs17780195, rs1558535, and rs4432291 were selected for our study.

National Institutes of Health (NIH) LDlink was accessed to calculate the linkage disequilibrium values of the five LINC00511 SNPs via linkage disequilibrium tool (LD Pair Tool) according to 1000 genomes [51] at https://ldlink.nih.gov/?tab=ldpair (accessed on 29 June 2024).

### 4.7. Blood Samples

Five milliliters of blood were collected from controls and BC patients into Ethylenediaminetetraacetic acid (EDTA) anticoagulant vacutainers. They were stored at −80 °C until biochemical assessment.

### 4.8. Routine Biochemical Testing

Patients’ levels of hemoglobin, white blood cells, neutrophils, lymphocytes, serum creatinine, and serum urea were all recorded from patients’ files at the NCI, New Cairo, Egypt.

### 4.9. DNA Extraction from Whole Blood

DNA was extracted from whole blood samples using the DNA Extraction Kit (QIAamp DNA Blood Mini Kit) (Cat. No. 51104; Qiagen, Sigma-Aldrich, 19300 Germantown Rd. Germantown, MD 20874, United States).

### 4.10. Quantitation of Purified DNA

The extracted DNA’s purity and concentration were assessed using a Quawell UV-Vis spectrophotometer (San Jose, CA, USA). The quantity of DNA in the sample was assessed using absorbance at 260 nm. The purity of DNA was assessed using absorbance at 260/280 nm ratios (accepted at 1.80–2.00).

### 4.11. SNPs Genotyping

The TaqMan^®^ SNP genotyping assay was used to perform the genotyping for LINC00511 polymorphisms (rs11657109, rs9906859, rs17780195, rs1558535, and rs4432291) by using StepOne Plus™ qRT-PCR system (Applied Biosystems, LLC, Thermo Fisher Scientific, 850 Lincoln Centre Drive, Foster City, CA 94404, USA).

### 4.12. Statistical Analysis

Data was analyzed using Statistical Package for the Social Sciences (SPSS) v.23.0 software, SHEsis software (http://analysis.bio-x.cn/SHEsisMain.htm), R programming version 4.5.1, and Python (https://www.python.org/).

For comparing baseline characteristics between patients and controls, the Mann–Whitney U test was used for continuous data (age), and the chi-square test was used for categorical data. The chi-square test was also used to test the association between LINC00511 SNPs alleles and BC risk. A logistic regression and stratified analysis were used to explore the association between LINC00511 SNPs and BC susceptibility. Moreover, logistic regression analyses were used to test the associations of LINC00511 SNPs with the ER, PR, and HER-2 status of BC patients; tumor grade; tumor stage; lymph node metastasis; and BC subtypes. Haplotype analysis was performed by using SHEsis software to determine the combined effects of the five SNPs of LINC00511. By R programming, Multifactor Dimensionality Reduction (MDR) using a three-way split internal validation approach was used to identify interactions between SNPs that are associated with BC. In addition, post hoc epistasis analysis was performed after MDR to focus on identifying interactions among specific SNPs which were chosen by MDR for their potential association with BC. Since five SNPs were tested, Bonferroni correction was applied to adjust for multiple comparisons. The significance threshold was therefore set at *p* < 0.01 instead of *p* < 0.05.

## 5. Conclusions

LINC00511 SNP rs11657109 significantly increased the risk of BC in the Egyptian population, suggesting its potential as a diagnostic marker. LINC00511 SNP rs11657109 was linked to a high risk of luminal A BC. LINC00511 SNP rs17780195 was associated with increased risk of luminal B BC, while LINC00511 SNP rs9906859 was associated with decreased luminal B BC risk. Study results suggest that LINC00511 SNP rs11657109 may be associated with BC susceptibility in the Egyptian population.

## Figures and Tables

**Table 1:** The association between LINC00511 SNPs and BC susceptibility using different genetic models.

SNP	Genetic Model	Genotype	Cases (%)	Controls (%)	*p* *	Adjusted OR (95%CI)
**rs11657109**	**Codominant**	**AA**	66 (24.7)	55 (36.7)		1
		**AT**	120 (44.9)	64 (42.7)	0.062	1.562 (0.977–2.498)
		**TT**	81 (30.3)	31 (20.7)	0.005	2.177 (1.260–3.763)
	**Dominant**	**AA**	66 (24.7)	55 (36.7)		1
		**AT + TT**	201 (75.3)	95 (63.3)	0.01	1.763 (1.143–2.719)
	**Recessive**	**AA + AT**	186 (69.7)	119 (79.3)		1
		**TT**	81 (30.3)	31 (20.7)	0.033	1.672 (1.041–2.684)
	**Over-dominant**	**AA + TT**	147 (55.1)	86 (57.3)		1
		**AT**	120 (44.9)	64 (42.7)	0.653	1.097 (0.733–1.642)
**rs9906859**	**Codominant**	**CC**	114 (42.7)	55 (36.7)		1
		**CT**	105 (39.3)	55 (36.7)	0.725	0.921 (0.582–1.456)
		**TT**	48 (18)	40 (26.7)	0.043	0.579 (0.341–0.982)
	**Dominant**	**CC**	114 (42.7)	55 (36.7)		1
		**CT + TT**	153 (57.3)	95 (63.3)	0.229	0.777 (0.515–1.172)
	**Recessive**	**CC + CT**	219 (82)	110 (73.3)		1
		**TT**	48 (18)	40 (26.7)	0.038	0.603 (0.374–0.972)
	**Over-dominant**	**CC + TT**	162 (60.7)	95 (63.3)		1
		**CT**	105 (39.3)	55 (36.7)	0.592	1.120 (0.741–1.692)
**rs17780195**	**Codominant**	**AA**	136 (50.9)	87 (58)		1
		**AG**	106 (39.7)	52 (34.7)	0.223	1.304 (0.851–1.999)
		**GG**	25 (9.4)	11 (7.3)	0.334	1.454 (0.681–3.104)
	**Dominant**	**AA**	136 (50.9)	87 (58)		1
		**AG + GG**	131 (49.1)	63 (42)	0.166	1.330 (0.889–1.991)
	**Recessive**	**AA + AG**	242 (90.6)	139 (92.7)		1
		**GG**	25 (9.4)	11 (7.3)	0.48	1.305 (0.623–2.734)
	**Over-dominant**	**AA + GG**	161 (60.3)	98 (65.3)		1
		**AG**	106 (39.7)	52 (34.7)	0.310	1.241 (0.818–1.881)
**rs1558535**	**Codominant**	**AA**	60 (22.5)	43 (28.7)		1
		**AT**	134 (50.2)	64 (42.7)	0.106	1.501 (0.917–2.454)
		**TT**	73 (27.3)	43 (28.7)	0.479	1.217 (0.707–2.095)
	**Dominant**	**AA**	60 (22.5)	43 (28.7)		1
		**AT + TT**	207 (77.5)	107 (71.3)	0.160	1.386 (0.879–2.187)
	**Recessive**	**AA + AT**	194 (72.7)	107 (71.3)		1
		**TT**	73 (27.3)	43 (28.7)	0.772	0.936 (0.600–1.461)
	**Over-dominant**	**AA + TT**	133 (49.8)	86 (57.3)		1
		**AT**	134 (50.2)	64 (42.7)	0.14	1.354 (0.905–2.025)
**rs4432291**	**Codominant**	**GG**	93 (34.8)	49 (32.7)		1
		**AG**	124 (46.4)	67 (44.7)	0.914	0.975 (0.618–1.539)
		**AA**	50 (18.7)	34 (22.7)	0.369	0.775 (0.444–1.352)
	**Dominant**	**GG**	93 (34.8)	49 (32.7)		1
		**AG + AA**	174 (65.2)	101 (67.3)	0.654	0.908 (0.594–1.387)
	**Recessive**	**GG + AG**	217 (81.3)	116 (77.3)		1
		**AA**	50 (18.7)	34 (22.7)	0.336	0.786 (0.481–1.284)
	**Over-dominant**	**GG + AA**	143 (53.6)	83 (55.3)		1
		**AG**	124 (46.4)	67 (44.7)	0.727	1.074 (0.719–1.605)

*p* * value of logistic regression analysis.

**Table 2:** Allele frequencies of the five SNPs in all the study subjects.

SNP	Alleles	Allele Frequency		*p* *	OR (95%CI)
		Cases (%)	Controls (%)		
rs11657109	A	47	58	0.003	1.545 (1.162–2.056)
	T	53	42		
rs9906859	C	62	55	0.038	0.738 (0.554–0.983)
	T	38	45		
rs17780195	A	71	75	0.159	1.26 (0.913–1.739)
	G	29	25		
rs1558535	A	48	5	0.5	1.102 (.831–1.463)
	T	52	5		
rs4432291	G	58	45	0.393	0.883 (0.664–1.174)
	A	42	55		

Two-sided χ2 test, *p* < 0.01 was considered statistically significant. *p* * value of logistic regression analysis.

**Table 3:** The association between BC molecular subtypes and LINC00511 SNPs, relative to controls.

SNP	Genetic Model of the SNP	Geno-Type	Controls	Luminal A BC	*p* *	OR (95%CI)	Luminal B BC	*p* *	OR (95%CI)	HER-2 BC	*p* *	OR (95%CI)	TNBC	*p* *	OR (95%CI)
			*n* = 150 *n* (%)	*n* = 175 *n* (%)			*n* = 64 *n* (%)			*n* = 16 *n* (%)			*n* = 12 *n* (%)		
**rs11657109**	**Codominant**	**AA**	55 (36.7)	38 (21.7)		1	16 (25)		1	7 (43.8)		1	5 (41.7)		1
		**AT**	64 (42.7)	80 (45.7)	0.028	1.809 (1.067–3.068)	29 (45.3)	0.220	1.558 (0.767–3.164)	5 (31.3)	0.427	0.614 (0.184–2.044)	6 (50)	0.691	1.031 (0.298–3.565)
		**TT**	31 (20.7)	57 (32.6)	0.011	2.661 (1.458–4.858)	19 (29.7)	0.067	2.107 (0.949–4.677)	4 (25)	0.984	1.014 (0.275–3.739)	1 (8.3)	0.354	0.355 (0.040–3.176)
	**Dominant**	**AA**	55 (36.7)	38 (21.7)		1	16 (25)		1	7 (43.8)		1	5 (41.7)		1
		**AT + TT**	95 (63.3)	137 (78.3)	0.003	2.087 (1.280–3.405)	48 (75)	0.099	1.737 (0.901–3.347)	9 (56.3)	0.579	0.744 (0.263–2.110)	7 (58.3)	0.730	0.811 (0.245–2.677)
	**Recessive**	**AA + AT**	119 (79.3)	118 (67.4)		1	45 (70.3)		1	12 (75)		1	11 (91.7)		1
		**TT**	31 (20.7)	57 (32.6)	0.017	1.854 (1.118–3.076)	19 (29.7)	0.155	1.621 (0.833–3.155)	4 (25)	0.687	1.280 (0.386–4.242)	1 (8.3)	0.322	0.349 (0.043–2.807)
	**Over-dominant**	**AA + TT**	86 (57.3)	95 (54.3)		1	35 (54.7)		1	11 (68.8)		1	6 (50)		1
		**AT**	64 (42.7)	80 (45.7)	0.581	1.132 (0.729–1.756)	29 (45.3)	0.721	1.113 (0.618–2.007)	5 (31.3)	0.382	0.611 (0.202–1.845)	6 (50)	0.623	1.344 (0.414–4.360)
**rs9906859**	**Codominant**	**CC**	55 (36.7)	78 (44.6)		1	27 (42.2)		1	4 (25)		1	5 (41.7)		1
		**CT**	55 (36.7)	64 (36.6)	0.437	0.821 (0.498–1.351)	28 (43.8)	0.912	1.037 (0.543–1.981)	8 (50)	0.280	2.000 (0.569–7.030)	5 (41.7)	1	1.000 (0.274–3.650)
		**TT**	40 (26.7)	33 (18.9)	0.065	0.582 (0.327–1.035)	9 (14.1)	0.074	0.458 (0.194–1.080)	4 (25)	0.666	1.375 (0.324–5.830)	2 (16.7)	0.488	0.550 (0.102–2.980)
	**Dominant**	**CC**	55 (36.7)	78 (44.6)		1	27 (42.2)		1	4 (25)		1	5 (41.7)		1
		**CT + TT**	95 (63.3)	97 (55.4)	0.149	0.720 (0.461–1.125)	37 (57.8)	0.447	0.793 (0.437–1.441)	12 (75)	0.359	1.737 (0.534–5.648)	7 (58.3)	0.730	0.811 (0.245–2.677)
	**Recessive**	**CC + CT**	110 (73.3)	142 (81.1)		1	55 (85.9)		1	12 (75)		1	10 (83.3)		1
		**TT**	40 (26.7)	33 (18.9)	0.094	0.639 (0.378–1.079)	9 (14.1)	0.048	0.450 (0.204–0.994)	4 (25)	0.868	0.917 (0.279–3.007)	2 (16.7)	0.453	0.550 (0.115–2.619)
	**Over-dominant**	**CC + TT**	95 (63.3)	111 (63.4)		1	36 (56.3)		1	8 (50)		1	7 (58.3)		1
		**CT**	55 (36.7)	64 (36.6)	0.986	0.996 (0.633–1.566)	28 (43.8)	0.331	1.343 (0.741–2.436)	8 (50)	0.301	1.727 (0.614–4.861)	5 (41.7)	0.730	1.234 (0.374–4.075)
**rs17780195**	**Codominant**	**AA**	87 (58)	97 (55.4)		1	28 (43.8)		1	6 (37.5)		1	5 (41.7)		1
		**AG**	52 (34.7)	64 (36.6)	0.678	1.104 (0.692–1.760)	28 (43.8)	0.107	1.673 (0.894–3.130)	8 (50)	0.158	2.231 (0.733–6.788)	6 (50)	0.269	2.008 (0.584–6.907)
		**GG**	11 (7.3)	14 (8)	0.753	1.142 (0.492–2.647)	8 (12.5)	0.112	2.260 (0.827–6.176)	2 (12.5)	0.269	2.636 (0.473–14.706)	1 (8.3)	0.688	1.582 (0.169–14.811)
	**Dominant**	**AA**	87 (58)	97 (55.4)		1	28 (43.8)		1	6 (37.5)		1	5 (41.7)		1
		**AG + GG**	63 (42)	78 (44.6)	0.641	1.110 (0.715–1.725)	36 (56.3)	0.05	1.776 (1.100–3.206)	10 (62.5)	0.124	2.302 (0.795–6.662)	7 (58.3)	0.279	1.933 (0.587–6.371)
	**Recessive**	**AA + AG**	139 (92.7)	161 (92)		1	56 (87.5)		1	14 (87.5)		1	11 (91.7)		1
		**GG**	11 (7.3)	14 (8)	0.822	1.099 (0.483–2.499)	8 (12.5)	0.229	1.805 (0.690–4.725)	2 (12.5)	0.470	1.805 (0.363–8.975)	1 (8.3)	0.899	1.149 (0.136–9.736)
	**Over-dominant**	**AA + GG**	98 (65.3)	111 (63.4)		1	36 (56.3)		1	8 (50)		1	6 (50)		1
		**AG**	52 (34.7)	64 (36.6)	0.721	1.087 (0.689–1.714)	28 (43.8)	0.210	1.466 (0.806–2.664)	8 (50)	0.231	1.885 (0.669–5.311)	6 (50)	0.293	1.885 (0.579–6.136)
**rs1558535**	**Codominant**	**AA**	43 (28.7)	39 (22.3)		1	13 (20.3)		1	7 (43.8)		1	1 (8.3)		1
		**AT**	64 (42.7)	88 (50.3)	0.131	1.516 (0.884–2.601)	34 (53.1)	0.139	1.757 (0.833–3.708)	7 (43.8)	0.485	0.672 (0.220–2.052)	5 (41.7)	0.276	3.359 (0.379–29.764)
		**TT**	43 (28.7)	48 (27.4)	0.496	1.231 (0.677–2.237)	17 (26.6)	0.530	1.308 (0.566–3.019)	2 (12.5)	0.131	0.286 (0.056–1.454)	6 (50)	0.104	6.000 (0.693–51.964)
	**Dominant**	**AA**	43 (28.7)	39 (22.3)		1	13 (20.3)		1	7 (43.8)		1	1 (8.3)		1
		**AT + TT**	107 (71.3)	136 (77.7)	0.188	1.401 (0.848–2.315)	51 (79.7)	0.205	1.577 (0.780–3.189)	9 (56.3)	0.217	0.517 (0.181–1.475)	11 (91.7)	0.161	4.421 (0.554–35.295)
	**Recessive**	**AA + AT**	107 (71.3)	127 (72.6)		1	47 (73.4)		1	14 (87.5)		1	6 (50)		1
		**TT**	43 (28.7)	48 (27.4)	0.804	0.940 (0.579–1.528)	17 (26.6)	0.754	0.900 (0.466–1.738)	2 (12.5)	0.183	0.355 (0.077–1.631)	6 (50)	0.132	2.488 (0.760–8.144)
	**Over-dominant**	**AA + TT**	86 (57.3)	87 (49.7)		1	30 (46.9)		1	9 (56.3)		1	7 (58.3)		1
		**AT**	64 (42.7)	88 (50.3)	0.170	1.359 (0.876–2.108)	34 (53.1)	0.161	1.523 (0.846–2.742)	7 (43.8)	0.934	1.045 (0.370–2.955)	5 (41.7)	0.946	0.960 (0.291–3.163)
**rs4432291**	**Codominant**	**GG**	49 (32.7)	64 (36.6)		1	20 (31.3)		1	3 (18.8)		1	6 (50)		1
		**AG**	67 (44.7)	81 (46.3)	0.759	0.926 (0.565–1.516)	31 (48.4)	0.715	1.134 (0.579–2.220)	7 (43.8)	0.455	1.706 (0.420–6.932)	5 (41.7)	0.435	0.609 (0.176–2.112)
		**AA**	34 (22.7)	30 (17.1)	0.212	0.676 (0.365–1.251)	13 (20.3)	0.877	0.937 (0.411–2.135)	6 (37.5)	0.153	2.882 (0.674–12.329)	1 (8.3)	0.196	0.240 (0.028–2.086)
	**Dominant**	**GG**	49 (32.7)	64 (36.6)		1	20 (31.3)		1	3 (18.8)		1	6 (50)		1
		**AG + AA**	101 (67.3)	111 (63.4)	0.461	0.841 (0.531–1.332)	44 (68.8)	0.839	1.067(0.569–2.002)	13 (81.3)	0.263	2.102 (0.572–7.721)	6 (50)	0.230	0.485 (0.149–1.582)
	**Recessive**	**GG + AG**	116 (77.3)	145 (82.9)		1	51 (79.7)		1	10 (62.5)		1	11 (91.7)		1
		**AA**	34 (22.7)	30 (17.1)	0.213	0.706 (0.408–1.221)	13 (20.3)	0.703	0.870 (0.424–1.785)	6 (37.5)	0.194	2.047 (0.694–6.039)	1 (8.3)	0.271	0.310 (0.039–2.489)
	**Over-dominant**	**GG + AA**	83 (55.3)	94 (53.7)		1	33 (51.6)		1	9 (56.3)		1	7 (58.3)		1
		**AG**	67 (44.7)	81 (46.3)	0.770	1.067 (0.689–1.654)	31 (48.4)	0.612	1.164 (0.647–2.092)	7 (43.8)	0.944	0.964 (0.341–2.723)	5 (41.7)	0.841	0.885 (0.269–2.914)

*p* * value of logistic regression analysis.

**Table 4:** Results of haplotype analysis of the five SNPs in LINC00511.

Haplotype	Cases (%)	Controls (%)	χ2	*p* Value	OR (95%CI)
**A A A A T**	23.76	36.67	9.058	0.003	0.617 (0.450–0.846)
**T G T A C**	19.69	12.78	10.628	0.001	1.945 (1.298–2.915)
**T G T G C**	16.18	16.86	0.201	0.654	1.092 (0.744–1.603)
**A G T A C**	5.56	9.06	2.206	0.138	0.664 (0.386–1.143)
**A A A G T**	5.26	0	18.382	<0.001	NA
**T G A A C**	4.19	7.23	2.231	0.135	0.630 (0.342–1.160)
**T A A A C**	3.68	1.33	4.869	0.027	3.191 (1.077–9.453)
**A G T A T**	2.44	3.67	0.539	0.463	0.737 (0.326–1.669)
**A G T G C**	2.39	3.3	0.254	0.615	0.805 (0.346–1.873)
**A A T A T**	1.04	3.33	4.454	0.035	0.340 (0.119–0.970)

SNP sequence for the haplotype: rs11657109, rs4432291, rs1558535, rs17780195, and rs9906859.

## Data Availability

The original contributions presented in this study are included in the article and Supplementary Materials. Further inquiries can be directed to the corresponding authors.

## Supplementary Materials

The following supporting materials information can be downloaded at: https://www.mdpi.com/article/10.3390/ijms26199328/s1.

## List of Abbreviations

BC	Breast Cancer
BIRADS	Breast-Imaging Reporting and Data System
bp	Base Pair
CA15-3	Cancer Antigen 15-3
CEA	Carcinoembryonic Antigen
ceRNA	Competing Endogenous RNA
CI	Confidence Interval
dbSNP	SNP Database
DNA	Deoxyribonucleic acid
E2F1	E2F Transcription Factor 1
EDTA	Ethylenediaminetetraacetic Acid
ER	Estrogen Receptor
HER-2	Human Epidermal Growth Factor Receptor 2
IGSR	International Genome Sample Resource
IRB	Institutional Review Board
LINC00511	Long Intergenic Non-Coding RNA 00511
LincRNAs	Long intergenic non-coding RNAs
LncRNAs	Long non-coding RNAs
LNM	Lymph Node Metastasis
MAF	Minor Allele Frequency
miR	micro-RNA
MDR	Multifactor Dimensionality Reduction
MMP13	Matrix Metallopeptidase 13
MRI	Magnetic Resonance Imaging
mRNAs	Messenger RNAs
NCBI	National Center for Biotechnology information
NCI	National Cancer Institute
NIH	National Institutes of Health
ncRNAs	Non-coding RNAs
OR	Odds Ratio
PR	Progesterone Receptor
qPCR	Quantitative Real-time Polymerase Chain Reaction
RNA	Ribonucleic Acid
SNPs	Single Nucleotide Polymorphisms
SPSS	Statistical Package for the Social Sciences
TNBC	Triple-Negative BC
TNM	Tumor–Node–Metastasis
UV-Vis	Ultraviolet–Visible
