# Supplementary material for: Long Intergenic Non-Coding RNA 00511 (LINC00511) Genetic Variations and Haplotypes in Breast Cancer: A Case-Controlled Study and Bioinformatics Analysis
Source: Int J Mol Sci. 2025 Sep 24;26(19):9328. doi: 10.3390/ijms26199328 (PMC12525368; doi:10.3390/ijms26199328)
Supplement: Supplementary file 1 [file ijms-26-09328-s001.zip › Supplementary Tables (LINC00511 SNPS) .pdf]

Supplementary materials

Table S1. The top 10 upregulated genes from DGE analysis

Gene Name	logFC	AveExpr	t	P.Value	adj.P.Val	B
UBE2C	7.950243372	4.747089495	15.19916903	6.23904897858037E-33	3.53296546827078E-29	64.19881918
MYBL2	7.929923202	4.931040442	13.3897477	5.85485059113169E-28	5.85071775542031E-25	52.92438022
HOXA7	7.734532166	2.54012453	6.167697751	5.38204227075331E-09	5.88944299597124E-08	10.22963168
TOP2A	7.574449124	6.413890963	13.64840308	1.12804083469445E-28	1.59692980831578E-25	54.56264667
RRM2	7.528949201	5.446758485	16.37286682	4.20873922781954E-36	3.57490310010991E-32	71.44447463
PBK	7.457101903	4.470266665	14.26689351	2.22400981690643E-30	6.29691312826774E-27	58.38714443
SHOX2	7.423420062	3.008143419	10.05409987	9.20430682592471E-19	1.20279049506776E-16	32.02363484

DLGAP5	7.290331943	3.253783793	13.43391449	4.41899614816796E-28	4.69186916031733E-25	52.96016746
BIRC5	7.19476885	4.49679835	13.02280119	6.07572429183385E-27	5.43233706682492E-24	50.60101311
MMP9	7.041410385	4.307234703	6.825235214	1.69246120621619E-10	2.67954622285187E-09	13.40130064

Table S2. LINC00511 SNPs alleles, chromosome, functional consequence, and MAF info report.

	LINC00511 SNPs				
	**rs11657109**	**rs9906859**	**rs17780195**	**rs1558535**	**rs4432291**
**Allele**	**[A>T]**	**[C>T]**	**[A>G]**	**[A>T]**	**[G>A]**
**Allele Flanks**	TAAAGCAAGA<br>GGATCTTATT<br>ATCTCAGAGT<br>TCTGGAGGCT<br>AGAAGTTTAA<br>AACCAAGGTG<br>TCAGTGGGGC<br>CATACTCCCT<br>CCAAAGCCTC<br>TAGGAGAGAA<br>**[A/T]**TTTCCTT<br>GCCTCTTCCA<br>GCTCCTGGTG<br>CTTCCTGGCT<br>GATATGGTTT<br>GGCTTTGTCC	GACAGGGGTAAAAA<br>AGAAATGTCTACATG<br>CCCCTTCACCTAGTT<br>TGCTCTTCAAATGTT<br>AGAGACCAACTTAC<br>AATCCACCCATGAGA<br>ACGGTCTGGATT**[C/T**<br>**]**CTCTGTGGTAAGAT<br>AGATCATCCCAAGA<br>GAAAACCCTAAACA<br>TAGTGATATTTAAGA<br>GTCTCCAGTGAAAAG<br>TGGAGTCTCTCTCTA<br>AGTGCCCTACAGC	CTCCTCATCCGCCAC<br>AACAGGAGACAAAA<br>CTGAACCAGGGAGA<br>GTCCAGATGTGGAGA<br>CTGCATAGGGCAGGG<br>GCAGCACAAGGACC<br>AGAAGACAGGGGT**[A**<br>**/G]**AAAAAGAAATGTC<br>TACATGCCCCTTCAC<br>CTAGTTTGCTCTTCA<br>AATGTTAGAGACCAA<br>CTTACAATCCACCCA<br>TGAGAACGGTCTGGA<br>TTTCTCTGTGGT	AACAAAACAAAACA<br>AAACAAAACACAAA<br>GACAGTCACAGCAA<br>CTACTGAGACGGGA<br>AAGCTATCATGGCAT<br>GGTGTGCATGCCTGT<br>ATTTTAAAACAGAC**[**<br>**A/T]**GATACTTACAGA<br>CTATCTTCCGGCTAA<br>ACATTTTCCTAAGTT<br>TTGGAAGAAGTCTCA<br>ATAAATTGGAACAA<br>GTCTCAATTTTCCTG<br>CTCAGAATTCTCTG	TTTAAC<br>ACACCT<br>ATTATTT<br>ATCACC<br>TTTAGA<br>AGCAGA<br>TTTCTCT<br>GATGTA<br>TACTAT<br>GCACCC<br>AGCACT<br>GTACAG<br>AAGAGA<br>ATTCTG<br>AGCAGG

	CCACTCAAAT CTCATCTTGA ATTATAGCTC CCATAATTCC CAC				AGAATT GG**[G/A]** CCTTTTC CAGTAT ACTGAG ACTTCTT CCAAAA CTTAAG AAAATG TTTAGCT GGAAGA TAGTCT GTGTCA GTCTGTT TTAAAA TACAGG CATGCA CACTGT
**VIC/FAM**	A/T	C/T	A/G	A/T	A/G

**Chromosome**	17:72624975 (GRCh38)	17:72628141 (GRCh38)	17:72628050 (GRCh38)	17:72612450 (GRCh38)	17:72610903 (GRCh38)
**Canonical SPDI**	NC_000017.11:72624974:A:T	NC_000017.11:72628140:C:T	NC_000017.11:72628049:A:G	NC_000017.11:72612449:A:T	NC_000017.11:72610902:G:A
**Functional Consequence**	Intron variant	Intron variant	Intron variant	Intron variant	Intron variant
**MAF**	T=0.408	T=0.192	G=0.07	T=0.465	A=0.278

[LINC00511: Long intergenic non-coding RNA 00511; SNPs: Single Nucleotide Polymorphisms; MAF: Minor Allele Frequency, GRCh38: Genome Reference Consortium Human Build 38; SPDI: Sequence Position Deletion Insertion].

Table S3. The baseline characteristics of 267 BC patients and 150 cancer-free controls.

Variables	Cases (%)	Controls (%)	*P*	OR (95%CI)
	N=267	N=150		
**Age** [Median (IQR)]	50 (39-55)	44 (40-47)	NS*	
**Menopausal status**				
Pre-menopausal	126 (47.2)	107 (71.3)		1
Post-menopausal	141 (52.8)	43 (28.7)	**< 0.001#**	2.785 (1.815-4.271)
**No. of pregnancies**				
<1	17 (6.4)	23 (15.3)		1
1	18 (6.7)	36 (24)	NS#	0.676 (0.291-1.574)
2	56 (21)	54 (36)	NS#	1.403 (0.676-2.911)
3	78 (29.2)	30 (20)	**0.001#**	3.518 (1.653-7.485)
≥ 4	98 (36.7)	7 (4.7)	**< 0.001#**	18.941 (7.035-50.997)
**ER expression**				
Negative	55 (20.6)			
Positive	212 (79.4)			
**PR expression**				
Negative	43 (16.1)			
Positive	224 (83.9)			
**HER-2 expression**				
Negative	165 (61.8)			
Positive	102 (38.2)			

*Mann-Whitney U test. #Two-sided $\chi 2$ test, $P < 0.01$ was considered statistically significant.

Table S4. Stratification analysis of the relationship between LINC00511 SNPs and BC susceptibility using the codominant model

SNP	Geno-type	<50		≥ 50		Pre-menopausal		Post-menopausal		<3 Pregnancies		≥ 3 pregnancies	
		P^*	OR (95%CI)	P^*	OR (95%CI)	P^*	OR (95%CI)	P^*	OR (95%CI)	P^*	OR (95%CI)	P^*	OR (95%CI)
**rs11657109**	AA		1		1		1		1		1		1
	AT	0.096	1.654 (0.915-2.990)	0.798	1.123 (0.462-2.728)	0.124	1.601 (0.879-2.917)	0.511	1.329 (0.569-3.103)	**0.005**	2.742 (1.353-5.555)	0.998	0.999 (0.452-2.205)
	TT	0.034	2.073 (1.055-4.074)	0.171	2.172 (0.716-6.584)	**0.003**	3.004 (1.444-6.248)	0.859	1.084 (0.444-2.647)	**0.009**	2.827 (1.289-6.199)	0.071	2.756 (0.916-8.291)
**rs9906859**	CC		1		1		1		1		1		1
	CT	0.447	0.799 (0.448-1.424)	0.299	1.606 (0.657-3.922)	0.105	0.608 (0.333-1.110)	0.023	2.762 (1.150-6.632)	0.794	0.921 (0.496-1.711)	0.623	0.810 (0.350-1.874)
	TT	0.174	0.633 (0.327-1.224)	0.437	0.677 (0.254-1.809)	0.127	0.587 (0.296-1.164)	0.410	0.693 (0.289-1.658)	0.082	.0506 (0.235-1.091)	0.146	0.508 (0.204-1.267)
**rs17780195**	AA		1		1		1		1		1		1
	AG	0.602	1.154 (0.675-1.972)	0.291	1.551 (0.687-3.503)	0.587	1.167 (0.668-2.039)	0.596	1.216 (0.591-2.502)	0.067	1.740 (0.962-3.149)	0.941	0.973 (0.467-2.025)
	GG	0.564	1.314 (0.519-3.322)	0.419	1.912 (0.398-9.189)	0.358	1.592 (0.591-4.290)	0.898	1.083 (0.320-3.670)	0.331	1.610 (0.617-4.201)	0.258	3.316 (0.415-26.506)
**rs1558535**	AA		1		1		1		1		1		1
	AT	0.450	1.263 (0.689-2.314)	0.146	2.075 (0.776-5.547)	0.567	1.196 (0.648-2.208)	0.147	1.930 (0.794-4.694)	0.159	1.649 (0.822-3.307)	0.385	1.459 (0.622-3.423)
	TT	0.658	1.169 (0.586-2.332)	0.982	0.989 (0.372-2.629)	0.830	1.081 (0.533-2.190)	0.821	1.111 (0.447-2.760)	0.535	1.276 (0.591-2.755)	0.603	1.288 (0.497-3.338)
**rs4432291**	GG		1		1		1		1		1		1
	AG	0.341	0.756 (0.424-1.346)	0.101	2.094 (0.867-5.057)	0.214	0.684 (0.375-1.245)	0.060	2.135 (0.970-4.699)	0.460	0.791 (0.425-1.473)	0.584	1.255 (0.556-2.834)

AA	0.434	0.756 (0.374-1.526)	0.898	0.938 (0.349-2.518)	0.363	0.714 (0.345-1.476)	0.964	0.980 (0.397-2.416)	0.294	0.658 (0.301-1.438)	0.731	0.847 (0.328-2.188)

*P value of logistic regression analysis

Table S5. Stratification analysis of the relationship between LINC00511 SNPs and BC susceptibility using the dominant model

	**rs11657109 (AT+TT/AA)**		**rs9906859 (CT+TT/CC)**		**rs17780195 (AG+GG/AA)**		**rs1558535 (AT+TT/AA)**		**rs4432291 (AG+AA/GG)**	
	P*	**OR (95%CI)**	***P****	**OR (95%CI)**	***P****	**OR (95%CI)**	***P****	**OR (95%CI)**	***P****	**OR (95%CI)**
< 50	0.034	1.799 (1.045-3.099)	0.239	0.731 (0.434-1.231)	0.515	1.182 (0.714-1.958)	0.475	1.229 (0.698-2.162)	0.308	0.756 (0.441-1.296)
≥ 50	0.435	1.397 (0.604-3.232)	0.687	1.169 (0.547-2.500)	0.230	1.606 (0.741-3.481)	0.372	1.487 (0.622-3.552)	0.244	1.580 (0.732-3.410)
Pre-menopausal	0.017	1.974 (1.131-3.445)	0.068	0.600 (0.347-1.038)	0.424	1.238 (0.734-2.090)	0.622	1.155 (0.652-2.043)	0.201	0.693 (0.395-1.215)
Post-menopausal	0.615	1.218 (0.564-2.631)	0.202	1.564 (0.786-3.110)	0.617	1.190 (0.601-2.358)	0.309	1.520 (0.679-3.406)	0.165	1.633 (0.817-3.262)
< 3	**0.002**	2.773 (1.432-5.370)	0.313	0.749 (0.427-1.313)	0.058	1.713 (0.981-2.990)	0.224	1.497 (0.781-2.869)	0.326	0.748 (0.419-1.334)
≥ 3	0.416	1.365 (0.645-2.890)	0.309	0.677 (0.320-1.435)	0.758	1.119 (0.548-2.287)	0.409	1.393 (0.634-3.060)	0.798	1.102 (0.523-2.320)

*P value of logistic regression analysis

Table S6. Stratification analysis of the relationship between LINC00511 SNPs and BC susceptibility using the recessive model

	rs11657109 (AA+AT/TT)		rs9906859 (CC+CT/TT)		rs17780195 (AA+AG/GG)		rs1558535 (AA+AT/TT)		rs4432291 (GG+AG/AA)	
	P*	**OR (95%CI)**	***P****	**OR (95%CI)**	***P****	**OR (95%CI)**	***P****	**OR (95%CI)**	***P****	**OR (95%CI)**
**< 50**	0.138	1.554 (0.868-2.782)	0.254	0.710 (0.394-1.278)	0.639	1.241 (0.503-3.063)	0.980	1.007 (0.568-1.788)	0.715	0.892 (0.483-1.646)
**≥ 50**	0.150	2.016 (0.776-5.238)	0.212	0.556 (0.221-1.398)	0.550	1.599 (0.343-7.457)	0.238	0.622 (0.282-1.368)	0.357	0.652 (0.263-1.620)
**Pre-menopausal**	0.012	2.286 (1.202-4.348)	0.388	0.768 (0.422-1.398)	0.410	1.504 (0.570-3.967)	0.912	0.967 (0.533-1.754)	0.740	0.899 (0.481-1.682)
**Post-menopausal**	0.798	0.909 (0.437-1.889)	0.085	0.481 (0.209-1.106)	0.987	0.990 (0.305-3.212)	0.360	0.716 (0.350-1.465)	0.358	0.677 (0.294-1.557)
**< 3**	0.213	1.487 (0.796-2.777)	0.076	0.527 (0.259-1.070)	0.610	1.272 (0.505-3.202)	0.785	0.918 (0.497-1.696)	0.422	0.751 (0.373-1.511)
**≥ 3**	0.046	2.758 (1.019-7.466)	0.162	0.566 (0.255-1.257)	0.249	3.354 (0.429-26.217)	0.976	1.012 (0.456-2.248)	0.492	0.745 (0.322-1.724)

*P value of logistic regression analysis

Table S7. Stratification analysis of the relationship between LINC00511 SNPs and BC susceptibility using the over-dominant model

	rs11657109 (AA+TT/AT)		rs9906859 (CC+TT/CT)		rs17780195 (AA+GG/AG)		rs1558535 (AA+TT/AT)		rs4432291 (GG+AA/AG)	
	P*	**OR (95%CI)**	***P****	**OR (95%CI)**	***P****	**OR (95%CI)**	***P****	**OR (95%CI)**	***P****	**OR (95%CI)**
**Age**										
**< 50**	0.491	1.196 (0.719-1.987)	0.868	0.957 (0.572-1.601)	0.690	1.112 (0.660-1.874)	0.539	1.170 (0.708-1.935)	0.512	0.845 (0.511-1.397)
**≥ 50**	0.522	0.780 (0.365-1.668)	0.169	1.799 (0.779-4.158)	0.378	1.432 (0.644-3.182)	0.070	2.088 (0.942-4.631)	0.067	2.140 (0.949-4.828)
**Menopausal status**										
**Pre-menopausal**	0.937	1.021 (0.608-1.716)	0.305	0.759 (0.449-1.284)	0.722	1.104 (0.640-1.904)	0.588	1.154 (0.688-1.934)	0.359	0.785 (0.468-1.316)
**Post-menopausal**	0.499	1.271 (0.634-2.548)	**0.009**	3.057 (1.322-7.068)	0.607	1.200 (0.598-2.407)	0.097	1.812 (0.899-3.652)	0.040	2.150 (1.036-4.460)
**No. of pregnancies**										
**< 3**	0.092	1.614 (0.924-2.819)	0.631	1.149 (0.651-2.030)	0.101	1.611 (0.910-2.851)	0.192	1.446 (0.830-2.520)	0.769	0.920 (0.528-1.603)
**≥ 3**	0.272	0.671 (0.330-1.367)	0.877	1.059 (0.511-2.198)	0.729	0.880 (0.427-1.815)	0.494	1.283 (0.628-2.621)	0.427	1.339 (0.652-2.751)

* *P* value of logistic regression analysis

Table S8. The associations of LINC00511 SNPs with ER, PR and HER-2 status of BC patients.

SNP	Genetic model of the SNP	Genotype	ER (-ve) N=55 N (%)	ER (+ve) N=212 N (%)	P^*	OR (95%CI)	PR (-ve) N=43 N (%)	PR (+ve) N=224 N (%)	P^*	OR (95%CI)	HER-2 (-ve) N=165 N (%)	HER-2 (+ve) N=102 N (%)	P^*	OR (95%CI)
**rs11657109**	**Codominant**	**AA**	18 (32.7)	48 (22.6)		1	16 (37.2)	50 (22.3)		1	42 (25.5)	24 (23.5)		1
		**AT**	23 (41.8)	97 (45.8)	0.204	1.582 (0.780-3.208)	17 (39.5)	103 (46)	0.088	1.939 (0.905-4.153)	76 (46.1)	44 (43.1)	0.967	1.013 (0.543-1.891)
		**TT**	14 (25.5)	67 (31.6)	0.147	1.795 (0.814-3.957)	10 (23.3)	71 (31.7)	0.064	2.272 (0.953-5.418)	47 (28.5)	34 (33.3)	0.489	1.266 (0.649-2.469)
	**Dominant**	**AA**	18 (32.7)	48 (22.6)		1	16 (37.2)	50 (22.3)		1	42 (25.5)	24 (23.5)		1
		**AT+TT**	37 (67.3)	164 (77.4)	0.125	1.662 (0.869-3.180)	27 (62.8)	174 (77.7)	0.041	2.062 (1.031-4.127)	123 (74.5)	78 (76.5)	0.723	1.110 (0.624-1.975)
	**Recessive**	**AA+AT**	41 (74.5)	145 (68.4)		1	33 (76.7)	153 (68.3)		1	118 (71.5)	68 (66.7)		1

		**TT**	14 (25.5)	67 (31.6)	0.378	1.353 (0.691-2.650)	10 (23.3)	71 (31.7)	0.273	1.531 (0.715-3.279)	47 (28.5)	34 (33.3)	0.403	1.255 (0.737-2.138)
	**Over-dominant model**	**AA+TT**	32 (58.2)	115 (54.2)		1	26 (60.5)	121 (54)		1	89 (53.9)	58 (56.9)		1
		**AT**	23 (41.8)	97 (45.8)	0.601	1.174 (0.644-2.138)	17 (39.5)	103 (46)	0.473	1.302 (0.669-2.533)	76 (46.1)	44 (43.1)	0.641	0.888 (0.540-1.461)
**rs9906859**	**Codominant**	**CC**	21 (38.2)	93 (43.9)		1	19 (44.2)	95 (42.4)		1	70 (42.4)	44 (43.1)		1
		**CT**	24 (43.6)	81 (38.2)	0.418	0.762 (0.395-1.470)	16 (37.2)	89 (39.7)	0.773	1.112 (0.539-2.297)	61 (37)	44 (43.1)	0.618	1.148 (0.668-1.971)
		**TT**	10 (18.2)	38 (17.9)	0.722	0.858 (0.37-1.992)	8 (18.6)	40 (17.9)	1	1 (0.405-2.472)	34 (20.6)	14 (13.7)	0.255	0.655 (0.316-1.356)
	**Dominant**	**CC**	21 (38.2)	93 (43.9)		1	19 (44.2)	95 (42.4)		1	70 (42.4)	44 (43.1)		1
		**CT+TT**	34 (61.8)	119 (56.1)	0.448	0.79 (0.43-1.451)	24 (55.8)	129 (57.6)	0.829	1.075 (0.557-2.075)	95 (57.6)	58 (56.9)	0.909	0.971 (0.59-1.6)
	**Recessive**	**CC+CT**	45 (81.8)	174 (82.1)		1	35 (81.4)	184 (82.1)		1	131 (79.4)	88 (86.3)		1

		**TT**	10 (18.2)	38 (17.9)	0.965	0.983 (0.455-2.122)	8 (18.6)	40 (17.9)	0.907	0.951 (0.41-2.205)	34 (20.6)	14 (13.7)	0.157	0.613 (0.311-1.208)
	**Over-dominant**	**CC+TT**	31 (56.4)	131 (61.8)		1	27 (62.8)	135 (60.3)		1	104 (63)	58 (56.9)		1
		**CT**	24 (43.6)	81 (38.2)	0.463	0.799 (0.438-1.456)	16 (37.2)	89 (39.7)	0.756	1.112 (0.567-2.182)	61 (37)	44 (43.1)	0.317	1.293 (0.782-2.14)
**rs17780195**	**Codominant**	**AA**	26 (47.3)	110 (51.9)		1	18 (41.9)	118 (52.7)		1	91 (55.2)	45 (44.1)		1
		**AG**	22 (40)	84 (39.6)	0.751	0.902 (0.478-1.703)	21 (48.8)	85 (37.9)	0.17	0.617 (0.31-1.229)	62 (37.6)	44 (43.1)	0.178	1.435 (0.848-2.492)
		**GG**	7 (12.7)	18 (8.5)	0.315	0.608 (0.23-1.607)	4 (9.3)	21 (9.4)	0.712	0.801 (0.246-2.603)	12 (7.3)	13 (12.7)	0.075	2.191 (0.925-5.188)
	**Dominant**	**AA**	26 (47.3)	110 (51.9)		1	18 (41.9)	118 (52.7)		1	91 (55.2)	45 (44.1)		1
		**AG+GG**	29 (52.7)	102 (48.1)	0.542	0.831 (0.459-1.506)	25 (58.1)	106 (47.3)	0.196	0.647 (0.334-1.252)	74 (44.8)	57 (55.9)	0.08	1.558 (0.948-2.56)
	**Recessive**	**AA+AG**	48 (87.3)	194 (91.5)		1	39 (90.7)	203 (90.6)		1	153 (92.7)	89 (87.3)		1

		**GG**	7 (12.7)	18 (8.5)	0.34	0.636 (0.251-1.61)	4 (9.3)	21 (9.4)	0.988	1.009 (0.328-3.1)	12 (7.3)	13 (12.7)	0.141	1.862 (0.815-4.258)
	**Over-dominant**	**AA+GG**	33 (60)	128 (60.4)		1	22 (51.2)	139 (62.1)		1	103 (62.4)	58 (56.9)		1
		**AG**	22 (40)	84 (39.6)	0.959	0.984 (0.537-1.804)	21 (48.8)	85 (37.9)	0.183	0.641 (0.332-1.235)	62 (37.6)	44 (43.1)	0.367	1.26 (0.762-2.084)
**rs1558535**	**Codominant**	**AA**	17 (30.9)	43 (20.3)		1	8 (18.6)	52 (23.2)		1	38 (23)	22 (21.6)		1
		**AT**	26 (47.3)	108 (50.9)	0.169	1.642 (0.811-3.327)	20 (46.5)	114 (50.9)	0.771	0.877 (0.363-2.121)	79 (47.9)	55 (53.9)	0.565	1.203 (0.642-2.253)
		**TT**	12 (21.8)	61 (28.8)	0.102	2.01 (0.871-4.635)	15 (34.9)	58 (25.9)	0.277	0.595 (0.233-1.517)	48 (29.1)	25 (24.5)	0.771	0.9 (0.441-1.837)
	**Dominant**	**AA**	17 (30.9)	43 (20.3)		1	8 (18.6)	52 (23.2)		1	38 (23)	22 (21.6)		1
		**AT+TT**	38 (69.1)	169 (79.7)	0.095	1.758 (0.906-3.411)	35 (81.4)	172 (76.8)	0.508	0.756 (0.33-1.731)	127 (77)	80 (78.4)	0.781	1.088 (0.6-1.973)
	**Recessive**	**AA+AT**	43 (78.2)	151 (71.2)		1	28 (65.1)	166 (74.1)		1	117 (70.9)	77 (75.5)		1

		**TT**	12 (21.8)	61 (28.8)	0.304	1.448 (0.715-2.931)	15 (34.9)	58 (25.9)	0.228	0.652 (0.326-1.306)	48 (29.1)	25 (24.5)	0.415	0.791 (0.451-1.389)
	**Over-dominant**	**AA+TT**	29 (52.7)	104 (49.1)		1	23 (53.5)	110 (49.1)		1	86 (52.1)	47 (46.1)		1
		**AT**	26 (47.3)	108 (50.9)	0.628	1.158 (0.64-2.098)	20 (46.5)	114 (50.9)	0.599	1.192 (0.62-2.292)	79 (47.9)	55 (53.9)	0.338	1.274 (0.777-2.09)
**rs4432291**	**Codominant**	**GG**	16 (29.1)	77 (36.3)		1	16 (37.2)	77 (34.4)		1	60 (36.4)	33 (32.4)		1
		**AG**	27 (49.1)	97 (45.8)	0.404	0.747 (0.376-1.484)	19 (44.2)	105 (46.9)	0.709	1.148 (0.555-2.376)	76 (46.1)	48 (47.1)	0.627	1.148 (0.657-2.006)
		**AA**	12 (21.8)	38 (17.9)	0.331	0.658 (0.283-1.529)	8 (18.6)	42 (18.8)	0.854	1.091 (0.431-2.76)	29 (17.6)	21 (20.6)	0.444	1.317 (0.651-2.662)
	**Dominant**	**GG**	16 (29.1)	77 (36.3)		1	16 (37.2)	77 (34.4)		1	60 (36.4)	33 (32.4)		1
		**AG+AA**	39 (70.9)	135 (63.7)	0.317	0.719 (0.377-1.372)	27 (62.8)	147 (65.6)	0.721	1.131 (0.575-2.226)	105 (63.6)	69 (67.6)	0.504	1.195 (0.709-2.014)
	**Recessive**	**GG+AG**	43 (78.2)	174 (82.1)		1	35 (81.4)	182 (81.3)		1	136 (82.4)	81 (79.4)		1

	**AA**	12 (21.8)	38 (17.9)	0.51	0.783 (0.377-1.624)	8 (18.6)	42 (18.8)	0.982	1.01 (0.437-2.334)	29 (17.6)	21 (20.6)	0.54	1.216 (0.651-2.272)
**Over-dominant**	**GG+AA**	28 (50.9)	115 (54.2)		1	24 (55.8)	119 (53.1)		1	89 (53.9)	54 (52.9)		1
	**AG**	27 (49.1)	97 (45.8)	0.659	0.875 (0.483-1.584)	19 (44.2)	105 (46.9)	0.746	1.115 (0.578-2.149)	76 (46.1)	48 (47.1)	0.874	1.041 (0.635-1.707)

*P value of logistic regression analysis.

Table S9. The association between LINC00511 SNPs and tumor stage.

SNP	Genetic model	Genotype	Tumor stage		P^*	OR (95%CI)
			I, II (N= 130) N (%)	III, IV (N= 137) N (%)		
**rs11657109**	**Codominant**	**AA**	32 (24.6)	34 (24.8)		1
		**AT**	56 (43.1)	64 (46.7)	0.812	1.076 (0.590-1.963)
		**TT**	42 (32.3)	39 (28.5)	0.685	0.874 (0.456-1.675)
	**Dominant**	**AA**	32 (24.6)	34 (24.8)		1
		**AT+TT**	98 (75.4)	103 (75.2)	0.969	0.989 (0.567-1.725)
	**Recessive**	**AA+AT**	88 (67.7)	98 (71.5)		1
		**TT**	42 (32.3)	39 (28.5)	0.495	0.834 (0.495-1.406)
	**Over-dominant**	**AA+TT**	74 (56.9)	73 (53.3)		1
		**AT**	56 (43.1)	64 (46.7)	0.550	1.159 (0.715-1.878)
**rs9906859**	**Codominant**	**CC**	60 (46.2)	54 (39.4)		1
		**CT**	47 (36.2)	58 (42.3)	0.245	1.371 (0.805-2.334)
		**TT**	23 (17.7)	25 (18.2)	0.584	1.208 (0.615-2.372)
	**Dominant**	**CC**	60 (46.2)	54 (39.4)		1
		**CT+TT**	70 (53.8)	83 (60.6)	0.266	1.317 (0.810-2.142)
	**Recessive**	**CC+CT**	107 (82.3)	112 (81.8)		1

		**TT**	23 (17.7)	25 (18.2)	0.906	1.038 (0.556-1.940)
	**Over-dominant**	**CC+TT**	83 (63.8)	79 (57.7)		1
		**CT**	47 (36.2)	58 (42.3)	0.302	1.297 (0.792-2.122)
**rs17780195**	**Codominant**	**AA**	75 (57.7)	61 (44.5)		1
		**AG**	46 (35.4)	60 (43.8)	0.070	1.604 (0.961-2.675)
		**GG**	9 (6.9)	16 (11.7)	0.083	2.186 (0.903-5.290)
	**Dominant**	**AA**	75 (57.7)	61 (44.5)		1
		**AG+GG**	55 (42.3)	76 (55.5)	0.032	1.699 (1.047-2.758)
	**Recessive**	**AA+AG**	121 (93.1)	121 (88.3)		1
		**GG**	9 (6.9)	16 (11.7)	0.187	1.778 (0.756-4.179)
	**Over-dominant**	**AA+GG**	84 (64.6)	77 (56.2)		1
		**AG**	46 (35.4)	60 (43.8)	0.161	1.423 (0.869-2.330)
**rs1558535**	**Codominant**	**AA**	30 (23.1)	30 (21.9)		1
		**AT**	60 (46.2)	74 (54)	0.5	1.233 (0.670-2.270)
		**TT**	40 (30.8)	33 (24.1)	0.582	0.825 (0.416-1.636)

	**Dominant**	**AA**	30 (23.1)	30 (21.9)		1
		**AT+TT**	100 (76.9)	107 (78.1)	0.818	1.070 (0.602-1.901)
	**Recessive**	**AA+AT**	90 (69.2)	104 (75.9)		1
		**TT**	40 (30.8)	33 (24.1)	0.222	0.714 (0.416-1.226)
	**Over-dominant**	**AA+TT**	70 (53.8)	63 (46)		1
		**AT**	60 (46.2)	74 (54)	0.2	1.370 (0.847-2.218)
**rs4432291**	**Codominant**	**GG**	46 (35.4)	47 (34.3)		1
		**AG**	57 (43.8)	67 (48.9)	0.610	1.150 (0.671-1.971)
		**AA**	27 (20.8)	23 (16.8)	0.605	0.834 (0.419-1.660)
	**Dominant**	**GG**	46 (35.4)	47 (34.3)		1
		**AG+AA**	84 (64.6)	90 (65.7)	0.853	1.049 (0.634-1.735)
	**Recessive**	**GG+AG**	103 (79.2)	114 (83.2)		1
		**AA**	27 (20.8)	23 (16.8)	0.405	0.770 (0.415-1.426)
	**Over-dominant**	**GG+AA**	73 (56.2)	70 (51.1)		1
		**AG**	57 (43.8)	67 (48.9)	0.408	1.226 (0.757-1.985)

**P* value of logistic regression analysis.

Table S10. The association between LINC00511 SNPs and lymph node metastasis

SNP	Genetic model	Genotype	Lymph node metastasis No (N= 82) N (%)	Lymph node metastasis Yes (N= 185) N (%)	P^*	OR (95%CI)
**rs11657109**	**Codominant**	**AA**	25 (30.5)	41 (22.2)		1
		**AT**	32 (39)	88 (47.6)	0.114	1.677 (0.883-3.184)
		**TT**	25 (30.5)	56 (30.3)	0.373	1.366 (0.688-2.710)
	**Dominant**	**AA**	25 (30.5)	41 (22.2)		1
		**AT+TT**	57 (69.5)	144 (77.8)	0.147	1.540 (0.859-2.763)
	**Recessive**	**AA+AT**	57 (69.5)	129 (69.7)		1
		**TT**	25 (30.5)	56 (30.3)	0.972	0.990 (0.562-1.742)
	**Over-dominant**	**AA+TT**	50 (61)	97 (52.4)		1
		**AT**	32 (39)	88 (47.6)	0.196	1.418 (0.835-2.407)
**rs9906859**	**Codominant**	**CC**	37 (45.1)	77 (41.6)		1
		**CT**	31 (37.8)	74 (40)	0.639	1.147 (0.646-2.037)
		**TT**	14 (17.1)	34 (18.4)	0.681	1.167 (0.559-2.435)
	**Dominant**	**CC**	37 (45.1)	77 (41.6)		1
		**CT+TT**	45 (54.9)	108 (58.4)	0.594	1.153 (0.683-1.948)
	**Recessive**	**CC+CT**	68 (82.9)	151 (81.6)		1
		**TT**	14 (17.1)	34 (18.4)	0.798	1.094 (0.551-2.170
	**Over-dominant**	**CC+TT**	51 (62.2)	111 (60)		1
		**CT**	31 (37.8)	74 (40)	0.735	1.097 (0.643-1.872)
**rs17780195**	**Codominant**	**AA**	42 (51.2)	94 (50.8)		1
		**AG**	34 (41.5)	72 (38.9)	0.843	0.946 (0.548-1.634)
		**GG**	6 (7.3)	19 (10.3)	0.491	1.415 (0.527-3.798)
	**Dominant**	**AA**	42 (51.2)	94 (50.8)		1
		**AG+GG**	40 (48.8)	91 (49.2)	0.951	1.016 (0.604-1.710)
	**Recessive**	**AA+AG**	76 (92.7)	166 (89.7)		1
		**GG**	6 (7.3)	19 (10.3)	0.447	1.450 (0.557-3.776)
		**AA+GG**	48 (58.5)	113 (61.1)		1

	**Over-dominant**	**AG**	34 (41.5)	72 (38.9)	0.695	0.900 (0.530-1.527)
**rs1558535**	**Codominant**	**AA**	16 (19.5)	44 (23.8)		1
		**AT**	36 (43.9)	98 (53)	0.977	0.990 (0.498-1.970)
		**TT**	30 (36.6)	43 (23.2)	0.084	0.521 (0.249-1.090)
	**Dominant**	**AA**	16 (19.5)	44 (23.8)		1
		**AT+TT**	66 (80.5)	141 (76.2)	0.441	0.777 (0.409-1.477)
	**Recessive**	**AA+AT**	52 (63.4)	142 (76.8)		1
		**TT**	30 (36.6)	43 (23.2)	0.025	0.525 (0.299-0.923)
	**Over-dominant**	**AA+TT**	46 (56.1)	87 (47)		1
		**AT**	36 (43.9)	98 (53)	0.172	1.439 (0.853-2.428)
**rs4432291**	**Codominant**	**GG**	28 (34.1)	65 (35.1)		1
		**AG**	39 (47.6)	85 (45.9)	0.832	0.939 (0.524-1.682)
		**AA**	15 (18.3)	35 (18.9)	0.989	1.005 (0.475-2.127)
	**Dominant**	**GG**	28 (34.1)	65 (35.1)		1
		**AG+AA**	54 (65.9)	120 (64.9)	0.876	0.957 (0.554-1.655)
	**Recessive**	**GG+AG**	67 (81.7)	150 (81.1)		1
		**AA**	15 (18.3)	35 (18.9)	0.904	1.042 (0.533-2.037)
	**Over-dominant**	**GG+AA**	43 (52.4)	100 (54.1)		1
		**AG**	39 (47.6)	85 (45.9)	0.807	0.937 (0.557-1.578)

*P value of logistic regression analysis

Table S11. The association between LINC00511 SNPs and tumor grade

SNP	Genetic model	Genotype	Tumor grade: Low (N= 203) N (%)	Tumor grade: High (N= 64) N (%)	P^{*}	OR (95%CI)
rs11657109	Codominant	AA	47 (23.2)	19 (29.7)		1
		AT	97 (47.8)	23 (35.9)	0.135	0.587 (0.291-1.182)
		TT	59 (29.1)	22 (34.4)	0.827	0.922 (0.447-1.902)
	Dominant	AA	47 (23.2)	19 (29.7)		1
		AT+TT	156 (76.8)	45 (70.3)	0.292	0.714 (0.381-1.337)
	Recessive	AA+AT	144 (70.9)	42 (65.6)		1
		TT	59 (29.1)	22 (34.4)	0.421	1.278 (0.703-2.325)
	Over-dominant	AA+TT	106 (52.2)	41 (64.1)		1
		AT	97 (47.8)	23 (35.9)	0.098	0.613 (0.343-1.095)
rs9906859	Codominant	CC	89 (43.8)	25 (39.1)		1
		CT	76 (37.4)	29 (45.3)	0.330	1.358 (0.733-2.516)
		TT	38 (18.7)	10 (15.6)	0.877	0.937 (0.410-2.140)
	Dominant	CC	89 (43.8)	25 (39.1)		1
		CT+TT	114 (56.2)	39 (60.9)	0.501	1.218 (0.686-2.161)
	Recessive	CC+CT	165 (81.3)	54 (84.4)		1
		TT	38 (18.7)	10 (15.6)	0.575	0.804 (0.376-1.722)
	Over-dominant	CC+TT	127 (62.6)	35 (54.7)		1
		CT	76 (37.4)	29 (45.3)	0.262	1.385 (0.784-2.444)
rs17780195	Codominant	AA	104 (51.2)	32 (50)		1
		AG	80 (39.4)	26 (40.6)	0.857	1.056 (0.583-1.913)
		GG	19 (9.4)	6 (9.4)	0.959	1.026 (0.378-2.789)
	Dominant	AA	104 (51.2)	32 (50)		1
		AG+GG	99 (48.8)	32 (50)	0.864	1.051 (0.599-1.843)
	Recessive	AA+AG	184 (90.6)	58 (90.6)		1
		GG	19 (9.4)	6 (9.4)	0.997	1.002 (0.382-2.627)
	Over-dominant	AA+GG	123 (60.6)	38 (59.4)		1
		AG	80 (39.4)	26 (40.6)	0.862	1.052 (0.593-1.865)
rs1558535	Codominant	AA	46 (22.7)	14 (21.9)		1
		AT	98 (48.3)	36 (56.3)	0.603	1.207 (0.594-2.455)
		TT	59 (29.1)	14 (21.9)	0.559	0.780 (0.338-1.797)
	Dominant	AA	46 (22.7)	14 (21.9)		1
		AT+TT	157 (77.3)	50 (78.1)	0.896	1.046 (0.531-2.061)

	**Recessive**	**AA+AT**	144 (70.9)	50 (78.1)		1
		**TT**	59 (29.1)	14 (21.9)	0.262	0.683 (0.351-1.330)
	**Over-dominant**	**AA+TT**	105 (51.7)	28 (43.8)		1
		**AT**	98 (48.3)	36 (56.3)	0.267	1.378 (0.783-2.425)
**rs4432291**	**Codominant**	**GG**	74 (36.5)	19 (29.7)		1
		**AG**	89 (43.8)	35 (54.7)	0.190	1.532 (0.809-2.899)
		**AA**	40 (19.7)	10 (15.6)	0.951	0.974 (0.413-2.294)
	**Dominant**	**GG**	74 (36.5)	19 (29.7)		1
		**AG+AA**	129 (63.5)	45 (70.3)	0.323	1.359 (0.740-2.495)
	**Recessive**	**GG+AG**	163 (80.3)	54 (84.4)		1
		**AA**	40 (19.7)	10 (15.6)	0.467	0.755 (0.354-1.611)
	**Over-dominant**	**GG+AA**	114 (56.2)	29 (45.3)		1
		**AG**	89 (43.8)	35 (54.7)	0.131	1.546 (0.879-2.720)

**P* value of logistic regression analysis

Table S12. The association between LINC00511 SNPs and BC molecular subtypes "TNBC and Triple positive BC"

SNP	Genetic model	Genotype	TNBC (N=12) N (16%)	Triple positive BC (N=62) N (84%)	P^*	OR (95%CI)
**rs11657109**	**Codominant**	**AA**	5	16		1
		**AT**	6	29	0.545	1.510 (0.398-5.737)
		**TT**	1	17	0.146	5.312 (0.558-50.553)
	**Dominant**	**AA**	5	16		1
		**AT+TT**	7	46	0.271	2.054 (0.570-7.393)
	**Recessive**	**AA+AT**	11	45		1
		**TT**	1	17	0.188	4.156 (0.498-34.683)
	**Over-dominant**	**AA+TT**	6	33		1
		**AT**	6	29	0.838	0.879 (0.255-3.027)
**rs9906859**	**Codominant**	**CC**	5	25		1
		**CT**	5	28	0.869	1.120 (0.290-4.328)
		**TT**	2	9	0.909	0.9 (0.148-5.489)
	**Dominant**	**CC**	5	25		1
		**CT+TT**	7	37	0.931	1.057 (0.301-3.708)
	**Recessive**	**CC+CT**	10	53		1
		**TT**	2	9	0.848	0.849 (0.159-4.531)
	**Over-dominant**	**CC+TT**	7	34		1
		**CT**	5	28	0.824	1.153 (0.330-4.032)
**rs17780195**	**Codominant**	**AA**	5	26		1
		**AG**	6	28	0.871	0.897 (0.244-3.297)
		**GG**	1	8	0.712	1.538 (0.156-15.171)
	**Dominant**	**AA**	5	26		1
		**AG+GG**	7	36	0.986	0.989 (0.282-3.464)
	**Recessive**	**AA+AG**	11	54		1

		**GG**	1	8	0.66	1.63 (0.185-14.382)
	**Over-dominant**	**AA+GG**	6	34		1
		**AG**	6	28	0.758	0.824 (0.239-2.838)
**rs1558535**	**Codominant**	**AA**	1	13		1
		**AT**	5	32	0.536	0.492 (0.052-4.632)
		**TT**	6	17	0.182	0.218 (0.023-2.041)
	**Dominant**	**AA**	1	13		1
		**AT+TT**	11	49	0.326	0.343 (0.04-2.902)
	**Recessive**	**AA+AT**	6	45		1
		**TT**	6	17	0.13	0.378 (0.107-1.334)
	**Over-dominant**	**AA+TT**	7	30		1
		**AT**	5	32	0.53	1.493 (0.427-5.218)
**rs4432291**	**Codominant**	**GG**	6	20		1
		**AG**	5	29	0.231	3.9 (0.42-36.240)
		**AA**	1	13	0.41	1.74 (0.466-6.491)
	**Dominant**	**GG**	6	20		1
		**AG+AA**	6	42	0.245	2.1 (0.601-7.334)
	**Recessive**	**GG+AG**	11	49		1
		**AA**	1	13	0.326	2.918 (0.345-24.717)
	**Over-dominant**	**GG+AA**	7	33		1
		**AG**	5	29	0.745	1.23 (0.352-4.3)

**P* value of logistic regression analysis

Table S13. The association between LINC00511 SNPs and BC molecular subtypes "Luminal B and non-luminal B BC"

SNP	Genetic model	Genotype	Non-luminal B BC (N=203) N (%)	Luminal B BC (N=64) N (%)	P^*	OR (95%CI)
rs11657109	Codominant	AA	50	16		1
		AT	91	29	0.991	0.996 (0.494-2.008)
		TT	62	19	0.911	0.958 (0.447-2.052)
	Dominant	AA	50	16		1
		AT+TT	153	48	0.952	0.98 (0.512-1.877)
	Recessive	AA+AT	141	45		1
		TT	62	19	0.897	0.96 (0.52-1.774)
	Over-dominant	AA+TT	112	35		1
		AT	91	29	0.946	1.02 (0.58-1.793)
rs9906859	Codominant	CC	87	27		1
		CT	77	28	0.611	1.172 (0.636-2.159)
		TT	39	9	0.491	0.744 (0.32-1.729)
	Dominant	CC	87	27		1
		CT+TT	116	37	0.925	1.028 (0.582-1.815)
	Recessive	CC+CT	164	55		1
		TT	39	9	0.352	0.688 (0.313-1.511)
	Over-dominant	CC+TT	126	36		1
		CT	77	28	0.407	1.273 (0.72-2.249)
rs17780195	Codominant	AA	108	28		1
		AG	78	28	0.287	1.385 (0.76-2.521)
		GG	17	8	0.213	1.815 (0.711-4.635)
	Dominant	AA	108	28		1
		AG+GG	95	36	0.188	1.462 (0.83-2.573)
	Recessive	AA+AG	186	56		1
		GG	17	8	0.326	1.563 (0.641-3.813)
	Over-dominant	AA+GG	125	36		1
		AG	78	28	0.448	1.246 (0.706-2.202)
rs1558535	Codominant	AA	47	13		1
		AT	100	34	0.578	1.229 (0.594-2.543)

		**TT**	56	17	0.824	1.098 (0.484-2.491)
	**Dominant**	**AA**	47	13		1
		**AT+TT**	156	51	0.635	1.182 (0.592-2.358)
	**Recessive**	**AA+AT**	147	47		1
		**TT**	56	17	0.873	0.949 (0.503-1.791)
	**Over-dominant**	**AA+TT**	103	30		1
		**AT**	100	34	0.59	1.167 (0.665-2.049)
**rs4432291**	**Codominant**	**GG**	73	20		1
		**AG**	93	31	0.543	1.282 (0.575-2.861)
		**AA**	37	13	0.548	1.217 (0.641-2.308)
	**Dominant**	**GG**	73	20		1
		**AG+AA**	130	44	0.491	1.235 (0.677-2.254)
	**Recessive**	**GG+AG**	166	51		1
		**AA**	37	13	0.709	1.144 (0.565-2.316)
	**Over-dominant**	**GG+AA**	110	33		1
		**AG**	93	31	0.714	1.111 (0.633-1.95)

*P value of logistic regression analysis

Table S14. MDR fit with three-way split validation.

Levels	Best SNPs	Training Accuracy	Testing Accuracy	Validation Accuracy
**1**	SNP1	52.7%	57.4%	66.0%
**2**	SNP1 and SNP4	60.7%	63.5%	70.1%
**3**	SNP2, SNP4 and SNP5	65.0%	70.5%	72.1%

[SNP1 is rs11657109, SNP2 is rs4432291, SNP3 is rs1558535, SNP4 is rs17780195 and SNP5 is rs9906859].

Table S15. Linkage disequilibrium and correlation between LINC00511 studied SNPs.

		LINC00511 SNPs				
**LINC00511 SNPs**	**Statistics**	**rs11657109**	**rs9906859**	**rs17780195**	**rs1558535**	**rs4432291**
**rs11657109**	D'	-	0.99	0.72	0.6	0.29
	r^2	-	0.16	0.06	0.29	0.02
	χ^2	-	212.27	75.44	382.31	29.74
	P	**-**	**<0.0001**	**<0.0001**	**<0.0001**	**<0.0001**
**rs9906859**	D'	0.99	-	1	0.79	0.69
	r^2	0.16	-	0.02	0.13	0.3
	χ^2	212.27	-	23.52	170.01	394.96
	P	**<0.0001**	**-**	**<0.0001**	**<0.0001**	**<0.0001**
**rs17780195**	D'	0.72	1	-	0.74	0.69
	r^2	0.06	0.02	-	0.05	0.01
	χ^2	75.44	23.52	-	61.54	17.92
	P	**<0.0001**	**<0.0001**	-	**<0.0001**	**<0.0001**
**rs1558535**	D'	0.6	0.79	0.74	-	0.68
	r^2	0.29	0.13	0.05	-	0.16
	χ^2	382.31	170.01	61.54	-	206.58
	P	**<0.0001**	**<0.0001**	**<0.0001**	-	**<0.0001**
**rs4432291**	D'	0.29	0.69	0.69	0.68	-
	r^2	0.02	0.3	0.01	0.16	-
	χ^2	29.74	394.96	17.92	206.58	-
	P	**<0.0001**	**<0.0001**	**<0.0001**	**<0.0001**	-

[LINC00511: Long intergenic non-coding RNA 00511; SNPs: Single nucleotide polymorphisms; D': Normalized linkage disequilibrium; r^2: Correlation coefficient; χ^2: Chi square; *P*: *P* value].
